# A global survey identifies novel upstream components of the *Ath5 *neurogenic network

**DOI:** 10.1186/gb-2009-10-9-r92

**Published:** 2009-09-07

**Authors:** Marcel Souren, Juan Ramon Martinez-Morales, Panagiota Makri, Beate Wittbrodt, Joachim Wittbrodt

**Affiliations:** 1 Developmental Biology Unit, EMBL-Heidelberg, Meyerhofstrasse, Heidelberg, 69117, Germany; 2 Centro Andaluz de Biología del Desarrollo (CABD), CSIC-Universidad Pablo de Olavide, Carretera de Utrera Km1, Sevilla, 41013, Spain

## Abstract

**Background:**

Investigating the architecture of gene regulatory networks (GRNs) is essential to decipher the logic of developmental programs during embryogenesis. In this study we present an upstream survey approach, termed trans-regulation screen, to comprehensively identify the regulatory input converging on endogenous regulatory sequences.

**Results:**

Our dual luciferase-based screen queries transcriptome-scale collections of cDNAs. Using this approach we study the regulation of *Ath5*, the central node in the GRN controlling retinal ganglion cell (RGC) specification in vertebrates. The *Ath5 *promoter integrates the input of upstream regulators to enable the transient activation of the gene, which is an essential step for RGC differentiation. We efficiently identified potential *Ath5 *regulators that were further filtered for true positives by an *in situ *hybridization screen. Their regulatory activity was validated *in vivo *by functional assays in medakafish embryos.

**Conclusions:**

Our analysis establishes functional groups of genes controlling different regulatory phases, including the onset of *Ath5 *expression at cell-cycle exit and its down-regulation prior to terminal RGC differentiation. These results extent the current model of the GRN controlling retinal neurogenesis in vertebrates.

## Abstract 2

Regulators of vertebrate Ath5 expression were identified by high-throughput screening; extending the current gene regulatory model network controlling retinal neurogenesis.

## Background

Gene regulatory networks (GRNs) determine the animal body plan and cooperate to specify the different cell types of the organism. They have evolved to integrate and precisely control developmental programs. While changes in the periphery of the networks may lead to subtle changes in body plan morphology, the GRN core architecture around central nodes remains more conserved [1].

In the vertebrate retina, the control of retinal progenitor cell (RPC) fate-choice and differentiation depends on the synchronization of intrinsic genetic programs and extrinsic signals. A hierarchical GRN controls the sequential generation of the different retinal cell types during embryogenesis [2]. There is increasing evidence that timing of cell cycle exit and cell-fate choice are closely linked, as cells forced to exit the cell cycle prematurely were more likely to adopt an early cell fate and vice versa [3-6]. The position of RPC nuclei within the developing neuroretina depends on the phase of the cell cycle. S-phase takes places at the basal side of the epithelium, while M-phase nuclei are located at the apical side [7-9].

**Figure 1:**
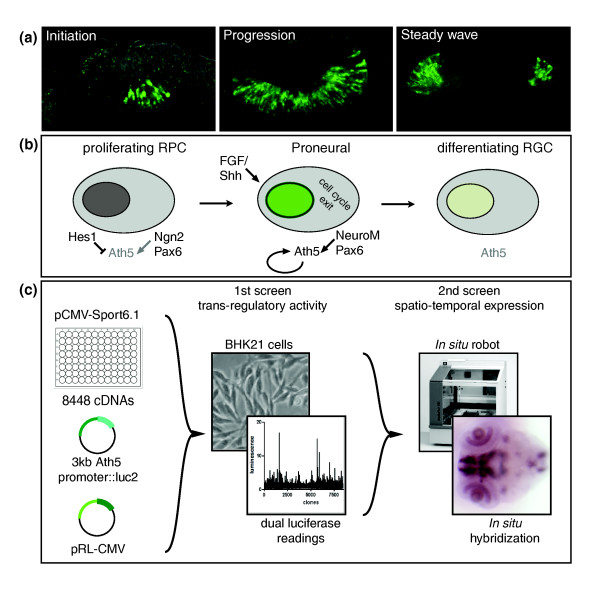
Screen overview. **(a) **Neurogenic wave in medaka. Single confocal sections through eye stained for *Ath5 *mRNA at the level of the lens. The sections show the neurogenic wave during its initiation, progression and steady wave stage. **(b) **Current model of *Ath5 *regulation. Three stages of *Ath5 *regulation have been identified: initial repression in proliferating RPCs; activation and maintenance in the proneural state around the exit of cell cycle by Fgf8, NeuroD, Pax6, and Ath5 itself; and finally terminal downregulation in differentiating RGCs. **(c) **Schematic overview of transregulation screen. We individually cotransfected 8,448 *Oryzias latipes *cDNAs with pGL3 *Ath5::Luc *and a cytomegalovirus (CMV)-driven *Renilla *luciferase control vector (pRL-CMV) into BHK21 cells in 96-well plates. Each transfection was carried out in triplicate. Identified candidates were filtered using semi-automated *in situ *hybridization. FGF, fibroblast growth factor; Shh, Sonic hedgehog.

In all vertebrate species analyzed, retinal ganglion cells (RGCs) are the first to be generated within an otherwise undifferentiated epithelium. The basic helix-loop-helix (bHLH) transcription factor *Ath5 *is the central switch in the GRN governing RGC neurogenesis. Loss of *Ath5 *in mouse and zebrafish leads to a complete absence of RGCs and an increase of later born cell types, such as amacrine cells and cone photoreceptors [10-12]. Gain-of-function experiments in chicken and frog showed that Ath5 promotes RGC formation at the expense of other cell types [13,14]. The onset of *Ath5 *expression in newborn RGCs coincides with the exit from the cell cycle [15,16]. RGCs are specified in a neurogenic wave that spreads across the retina similar to the morphogenetic furrow that moves through the eye imaginal disc in *Drosophila *[17]. RGCs first appear ventro-nasally close to the optic stalk in zebrafish [18,19]. Subsequently, a wave of differentiating cells spreads to the periphery of the eye [20-22]. In medaka, newborn RGCs first appear in the center of the retina at the initiation stage (IS). During the progression stage (PS), neuronal differentiation proceeds towards the peripheral retina. The final stage is a 'steady wave stage' (SWS) in which newborn RGCs are found exclusively in a ring in the peripheral ciliary marginal zone. At this stage retinal progenitor cells derived from the ciliary marginal zone undergo neurogenesis and contribute to the layered structure of the central retina (Figure 1a).

The initiation of *Ath5 *expression and RGC differentiation depends on extra-cellular signals emanating from the optic stalk [19]. Extra-cellular signals involved in RGC formation include members of the Wnt and fibroblast growth factor (FGF) signaling cascade [23,24]. Soluble molecules produced by RGCs themselves, such as Fgf19 and Sonic hedgehog (Shh), have been implicated in the spread of the wave [25,26]. However, the *Ath5 *promoter is activated in a wave-like manner even in the absence of RGCs in the zebrafish *Ath5 *mutant *lakritz*. Mutant cells initiate *Ath5 *expression according to their initial position when transplanted to a different spot in the retina [27]. These data support a cell-intrinsic mechanism triggering *Ath5 *expression. A small number of transcription factors have been shown to directly regulate *Ath5 *expression *in vivo *(Figure 1b). The bHLH factor *Hes1*, activated downstream of the Notch pathway, has been shown to repress the formation of RGCs and other cell types in mouse, such as rod photoreceptors and horizontal and amacrine cells prior to the onset of neurogenesis [28,29]. In chicken, Hes1 was shown to repress *Ath5 *in proliferating RPCs [30]. After the onset of *Ath5 *expression at the last mitosis, Ath5 protein binds to and activates its own promoter [31,32]. Additionally, it also receives positive regulatory input from Ngn2, NeuroM and Pax6 [33-36]. The terminal differentiation of RGCs is accompanied by a downregulation of *Ath5*, which is no longer expressed in mature neurons [30].

The *Ath5 *promoter integrates important upstream input to initiate RGC specification [2]. However, little is known about the transcriptional regulators governing the onset of *Ath5 *expression at the transition from proliferating progenitors to early post-mitotic cells and its downregulation prior to terminal differentiation. It is, for example, unclear how general cell cycle regulators may impinge upon the GRN controlling RGC specification.

The analysis of upstream gene regulation for key developmental genes has mainly focused on the dissection of the *cis*-regulatory logic using approaches such as promoter bashing or computational predictions. The systematic identification of *trans*-acting genes regulating a defined promoter has so far relied on binding assays such as yeast-one-hybrid assays [37]. Yeast-one-hybrid assays have been used to identify protein-DNA interactions based on the activity of a DNA-binding protein fused to an activating or repressing domain. Recently, the use of bacterial hybrid-screening technology and oligo arrays have overcome some of the limitations of the extensive cloning required [38,39], but these methods still depend on the generation of fusion proteins and only allow testing of a limited number of protein-DNA interactions. Initial attempts have been made to overcome these limitations by the use of luciferase-reporter based assays that employ synthetic reporter constructs [40].

Here, we present an upstream regulation survey, termed trans-regulation screen (TRS), using two nested screens to identify novel regulatory input on the *Ath5 *promoter (Figure 1c). The dual luciferase-based screening strategy allows surveying transcriptome-scale collections of full-length native cDNAs. They are tested for their activating or repressing properties on an endogenous promoter in vertebrate cells. The candidates were further filtered in a semi-automated *in situ *hybridization screen. Through this approach we have identified novel regulators of *Ath5*, and gained insight into the control of the retinal neurogenic network. Here we show the power of TRS technology as an upstream approach to survey developmental regulatory networks.

## Results

### The trans-regulation screen identifies candidate regulators of *Ath5*

To gain insight into the molecular mechanisms controlling the dynamic expression of *Ath5*, we explored the regulatory logic of a medakafish 3-kb promoter fragment that fully recapitulates the endogenous *Ath5 *expression pattern *in vivo *[31]. Using this promoter, we tested the ability of individual cDNAs to either activate or repress a luciferase reporter construct upon co-expression in BHK21 cells.

We employed a sequenced and arrayed medaka cDNA expression library, comprising unigene full-length clones in pCMV-Sport6, to individually test 8,448 genes. Our high-throughput trans-regulation screen allows efficient and reliable normalization using a second control reporter. We co-transfected each cDNA with the *Ath5 *firefly luciferase reporter (*Ath5::luc2*) and a cytomegalovirus (CMV)-driven *Renilla *luciferase control vector (pRL-CMV) in triplicate in a 96-well format. Luminescence levels of reporter and control were recorded after 48 h (Figure 1c). As a control we tested in parallel the known regulators of Ath5 - Hes-1, Pax6 and Ath5 itself - under screening conditions. We confirmed that Hes1 has a strong repressive activity on the 3-kb promoter fragment, while Pax6 and Ath5 can activate the promoter in a dose-dependent manner (Figure S1 in Additional data file 1) as previously reported [34-36].

**Figure 2:**
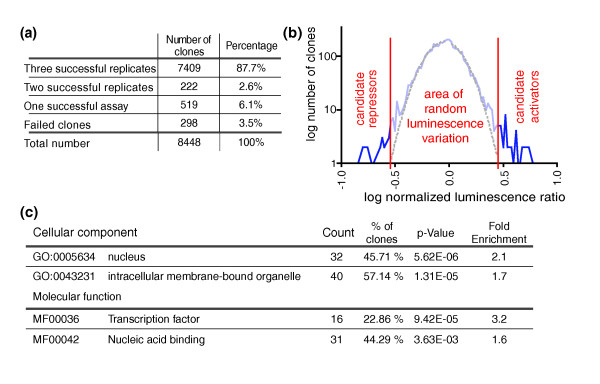
Screening statistics and candidate selection. **(a) **Screening statistics. The table lists the number of successful replicates per clone. **(b) **Selection of clones with non-random luminescence variation. All luminescence ratios were transformed into log-space for visualization. Luminescence ratios with a negative log value indicate a repressive effect, and positive log values an activating effect. The dotted line represents a Gaussian normal distribution fitted to the dataset. The left vertical line labels the threshold for repressors (less than 10^-0.544 ^= 0.2859), and the right vertical line labels the threshold for activators (more than 10^0.458 ^= 2.8732) **(c) **Gene Ontology analysis of candidate regulators. Candidates were analyzed for cellular localization and molecular function independently. The most abundant, non-redundant categories with a significant enrichment in the dataset compared to the genome are depicted.

The inclusion of the CMV-driven *Renilla *luciferase control [41] in the screen reduced the average standard deviation from 35.5 ± 80.2% to 17.3 ± 19.3% and was essential to correct for unspecific variation such as initial cell number, cell proliferation rate and transfection efficiency. As quality thresholds, we discarded those clones for which *Renilla *luminescence values were below 8,000 relative luminescence units, reflecting low cell numbers and/or general toxicity of the transfected construct. In addition, clones yielding firefly luminescence values smaller than ten times the background signal (ten raw units) were discarded. All raw luminescence readings were stored in a FileMaker database. Median values were calculated and normalized and statistics were generated using Prism software (supplementary material and methods in Additional data file 1). For 87.7% of the clones all three assays were successful, reflecting the robustness and the reliability of the screening setup (Figure 2a). To remove the plate-to-plate variation, we normalized each ratio (firefly over *Renilla*) against the average of all ratios in the plate. This approach has been previously employed [42] and was used as all plates are likely to only contain a very small number of regulators. Figure 2b represents the normalized ratios for all clones in a frequency distribution histogram in log-space.

As only a small number of cDNAs are likely to have an effect on the *Ath5 *promoter, the variation of luminescence ratios around the average can be regarded as random for almost all cDNAs while values outside a normal distribution curve are unlikely to be random variations. We therefore could fit a Gaussian normal distribution to the data (Figure 2b) and selected candidate genes based on mathematical criteria. Thus, clones with a normalized ratio of less than 0.2859 or more than 2.8732 were selected as candidates. In addition, only candidates with a standard deviation within the average standard deviation of all clones (15.7 ± 19.1%) were chosen. Ninety-three full-length cDNAs fulfill these criteria and can be mapped onto genes in the Ensembl gene build (Table S1 in Additional data file 1). They make up 1.1% of the total number of clones screened. Of these cDNAs, 28 are *in vitro *repressors, and 65 are *in vitro *activators. We analyzed the Gene Ontology terms associated with the candidates using the DAVID webtools (Figure 2c). Of all candidates with a GO annotation, 45.7% are localized in the nucleus and 44.3% are nucleic acid binding factors. The screening technology therefore gives a concise list of candidates that is enriched for nuclear factors involved in gene regulation.

### Nested *in situ *hybridization analysis refines the dataset to 53 high-confidence candidates

To assess whether the candidates can act as regulators *in vivo*, we determined their expression patterns. Using an *in situ *hybridization robot, we examined three different stages of development that coincide with the different phases of the *Ath5 *wave: initiation (IS, stage 24), progression (PS, stage 27) and steady wave stage (SWS, stage 31). All images of expression patterns have been submitted to the Medaka Expression Pattern Database [43] (Figure S2 in Additional data file 1). For 17 clones no expression was found at the tested stages and 23 genes were expressed in different domains of the embryo. Consistent with a function in *Ath5 *regulation, 10 genes were expressed ubiquitously at all time, while 43 genes were expressed specifically in the eye at one or more time points.

These specifically and dynamically expressed genes were analyzed by double fluorescence whole-mount *in situ *hybridization, using *Ath5 *as reference probe in parallel, to determine the exact relative expression patterns of *Ath5 *and the candidate regulators. According to their spatio-temporal expression, they were grouped into four categories (Table 1). Group 1 consists of 9 candidate repressors expressed in RPCs and early RGCs, group 2 of 25 candidate activators expressed in these cells. Group 3 contains three candidate activators expressed in late differentiating RGCs and group 4 contains six candidate repressors expressed in late differentiating RGCs.

**Table 1:** List of candidates

**Name**	**Fold-change**	**ID**
Group 1: repressors in RPCs		
AATF	0.21 ± 0.02	Rb-binding protein Che-1
ARG1	0.27 ± 0.02	Liver-type arginase
ATP-synthase	0.28 ± 0.04	ATP synthase beta chain
Cnot10	0.15 ± 0.01	CCR4-NOT transcription complex, subunit 10
DuS4L	0.28 ± 0.01	tRNA-dihydrouridine synthase 4-like
KPNA4	0.25 ± 0.04	Importin alpha-4 subunit
MCM2	0.27 ± 0.00	DNA replication licensing factor 2
USP25	0.28 ± 0.05	Ubiquitin carboxyl-terminal hydrolase 25
WDR43	0.29 ± 0.04	WD repeat protein 43, unknown function
		
Group 2: activators in RPCs		
Bcat2	2.93 ± 0.27	Mitochondrial branched chain aminotransferase 2
Cbx7	2.96 ± 0.14	Polycomb group gene
CEB55	4.70 ± 0.39	Centrosomal protein of 55 kDa
GPI deacetylase	15.00 ± 0.81	Vesicular transport
PTPN2	3.65 ± 0.59	Tyrosine-protein phosphatase non-receptor
Rb1	3.50 ± 0.46	Cell cycle exit, transcription factor
SRP40	2.88 ± 0.10	Splicing factor
Sterol demethylase	3.33 ± 0.33	Sterols and steroids biosynthesis, oocyte maturation
Thiolase	4.98 ± 0.32	Trifunctional enzyme, acetyl-CoA transferase
TMEM79	3.01 ± 0.30	Transmembrane protein, function unclear
TMP49	3.92 ± 0.42	Transmembrane protein, function unknown
Transferase	3.39 ± 0.58	Arginine n-methyl-transferase
Bub3	4.85 ± 0.00	Mitotic checkpoint protein
FAN	3.28 ± 0.28	Associated with N-SMase activation
Hsp1	11.02 ± 1.36	Heat shock protein 1
KPNA2	3.65 ± 0.36	Importin alpha-2 subunit,
MCM3	3.00 ± 0.00	DNA replication licensing factor 3
MRPL47	3.33 ± 0.00	Mitochondrial ribosomal protein L47 isoform b
NHL-domain II	3.36 ± 0.59	NHL-domain containing, unknown function
Ribonuclease	4.15 ± 0.09	Ribonuclease HI large subunit
sFRP-1	2.97 ± 0.55	Wnt-signal regulator
TARBP2	3.05 ± 0.18	TAR RNA-binding protein 2
Tetraspanin-9	3.29 ± 0.00	Transmembrane protein, interacts with integrins
USP1	3.25 ± 0.34	Ubiquitin carboxyl-terminal hydrolase 1
		
Group 3: activators in RGCs		
Ndrg3a	3.73 ± 0.00	N-myc downstream regulated 3, function unknown
Islet2	5.11 ± 0.22	Insulin gene enhancer, transcription factor
Tetraspanin-31	3.00 ± 0.38	Transmembrane protein, unknown function
		
Group 4: repressors in RGCs		
ELG protein	0.27 ± 0.00	mRNP complex, unknown function
Idax	0.16 ± 0.01	Negative regulation of Wnt signaling
NHL-protein	0.27 ± 0.00	NHL-domain containing, unknown function
RBM4L	0.23 ± 0.01	RRM-class RNA-binding protein
RBPMS2	0.20 ± 0.06	RNA-binding protein RNP-1, unknown function
Zfp 161	0.23 ± 0.01	Zinc finger, function unclear
		
Ubiquitously expressed regulators		
HMG	2.93 ± 0.49	HMG box DNA-binding domain
p65 TF	6.16 ± 0.81	NF-κB transcription factor p65
Beta-actin	0.27 ± 0.03	Cytoskeleton
Tubulin alpha-1B chain	3.28 ± 0.59	Cytoskeleton
UBR2	3.18 ± .0.36	Ubiquitin-protein ligase E3 component N-recognin-2
Uncharacterized1	0.22 ± 0.01	Unknown function
Coiled-coil domain	3.25 ± 0.43	Unknown function
EF-1-alpha	3.31 ± 0.26	Elongation factor
Nfkbia	2.99 ± 0.44	NF-kappaB inhibitor
Ankrd39	5.40 ± 0.71	Ankyrin repeat domain-containing protein 39, unknown function

Candidate clones were selected based on their relative effect on the reporter construct. Out of this list, clones with a specific spatio-termporal expression in the eye were grouped into four categories (groups 1 to 4). An additional category contains clones expressed ubiquitously. For each clone the fold-change of reporter activity with standard deviation and a short description of the gene are shown.

**Figure 3:**
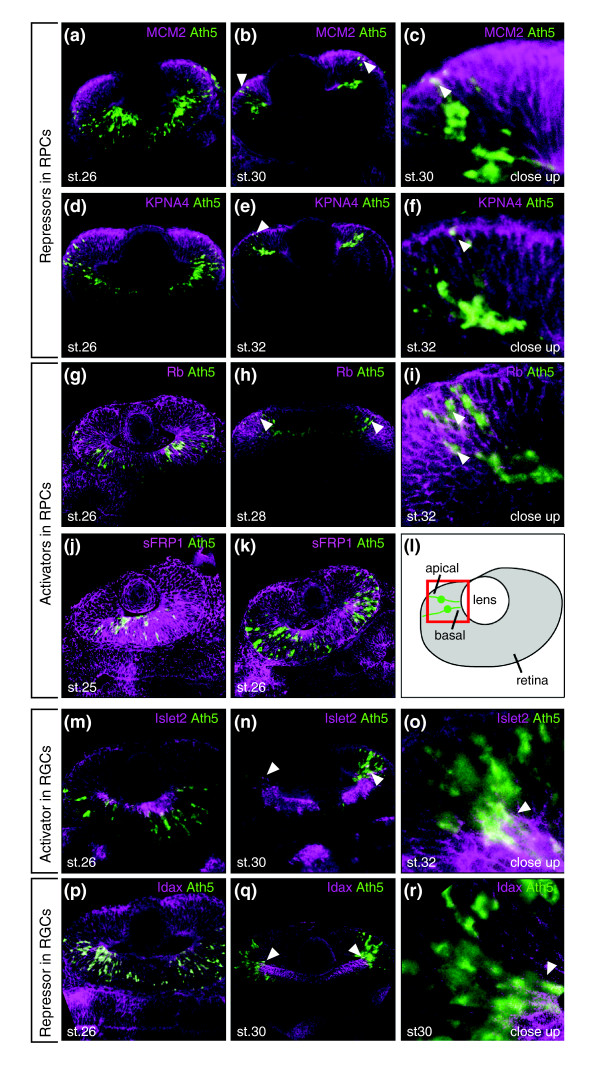
Double-fluorescent whole-mount *in situ *hybridization of candidates. *Ath5 *mRNA was detected using TSA-fluorescein (shown in green), and regulator mRNA was visualized using FastRed staining (shown in purple). **(a-f) **Group 1, repressors in RPCs. **(g-k) **Group 2, activators in RPCs. **(l) **A schematic representation of a SWS retina. The box demarcates the magnification shown in the close-ups of the transition zone of *Ath5 *and candidate regulator expression. **(m-o) **Group 3, activators in RGCs. **(p-r) **Group 3, repressors in RGCs. In this and subsequent figures, all images are single horizontal confocal sections of the developing eye at the level of the lens, anterior is to the left. Arrowheads point to sites of co-expression of *Ath5 *and the candidate regulator.

**Figure 4:**
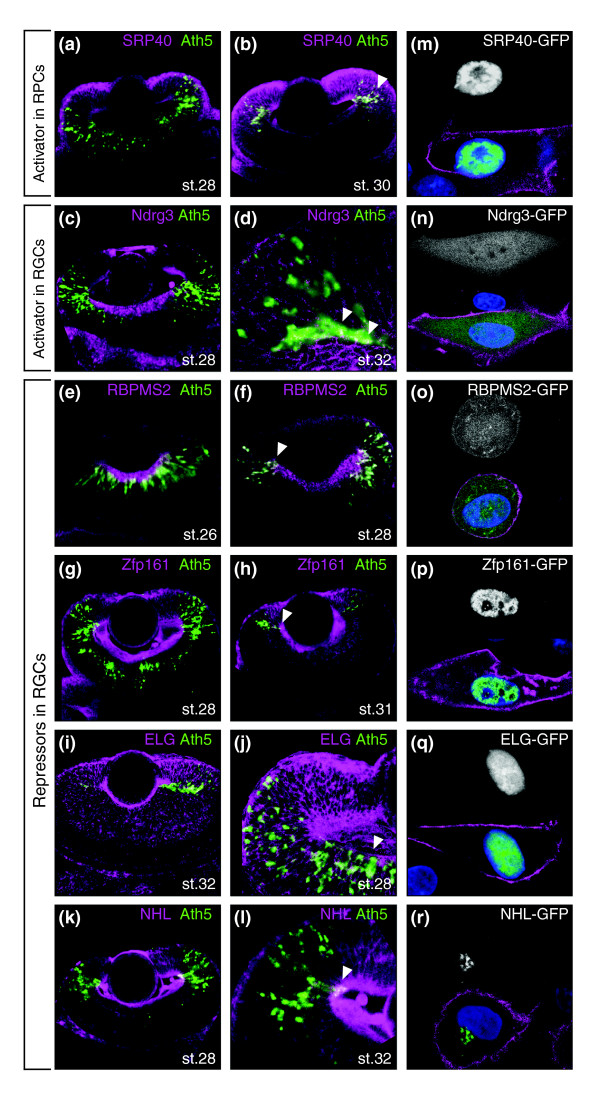
Double-fluorescent whole-mount *in situ *hybridization (DFWIS) of novel regulators and subcellular localization. DFWIS A-L. *Ath5 *mRNA was detected using TSA-fluorescein (green), and regulator mRNA was visualized using FastRed staining (purple). **(a, b) **Group 1, activators in RPCs. **(c, d) **Group 3, activators in RGCs. **(e-l) **Group 4, repressors in RGCs. **(m-r) **Cellular localization. BHK21 cells were transfected with GFP-fusion proteins. The upper half of each image shows the single channel including the GFP-fusion protein. The lower half of each image shows an overlay of the GFP-fusion protein (green), DAPI-stained nucleus (blue) and lynd-Tomato stained cell membrane (purple).

The expression of group 1 genes (repressors) becomes restricted to the retinal periphery as the neurogenic wave proceeds. Genes of this group overlap with *Ath5 *only in the early post-mitotic RGCs located apically in the differentiating epithelium (arrowheads in Figure 3c, f). They include replication complex factors MCM2 and 3 (Figure 3a-c; Figure S3a, b in Additional data file 1), the importin-family members KPNA4 and 2 (Figure 3d-f; Figure S3c in Additional data file 1), the regulator complex protein Cnot10 and a sterol demethylase (Figure S3d-f in Additional data file 1). Representative examples of group 2 (activators) are Retinoblastoma (Rb), secreted frizzled related protein (sFRP)1 and SRP40 (Figures 3g-k and 4a-c). *Rb *overlaps with *Ath5 *in apically located early RGCs (arrowheads in Figure 3h, i) exiting the cell cycle at all stages of the wave. *sFRP1 *is expressed at IS and PS, but ceases to be expressed at SWS (Figure 3j, k). SRP40, a splicing factor-like protein without known function, is found in RPCs and early RGCs at SWS (Figure 4a, b).

Group 3 genes (late activators) include Islet-2 (Figure 3m, n) and Ndrg3 (Figure 4c, d). Finally, group 4 (late repressors) includes Idax, a negative regulator of the Wnt-pathway (Figure 3p, q), the nucleotide-binding protein RBPMS2 (Figure 4e, f), the zinc-finger containing protein Zfp-161 (Figure 4g, h), ELG-protein (Figure 4i, j) and the novel NHL-domain containing protein (Figure 4k, l). Group 3 as well as group 4 genes are co-expressed with *Ath5 *only in a few terminally migrating RGCs located basally (arrowheads in Figures 3r and 4f, h, j, l) and maintain their expression in already localized RGCs.

These four categories define distinct regulatory activities at two critical points of *Ath5 *regulation, the onset of *Ath5 *expression in RPCs exiting the cell cycle and the sharp terminal downregulation in late migrating RGCs.

### Candidates that act dose-dependently are potential direct regulators of *Ath5*

We further characterized the activity of individual *in situ *validated candidates by assessing the dose-dependence of their regulatory effect. We employed our high-throughput pipeline to perform experiments for each candidate across a wide range of concentrations. Parallel experiments using CMV- and SV40-driven reporters were performed independently as a control to exclude regulatory effects on the reference promoters. We obtained data for 45 genes expressed in the eye. Of these, 19 exhibited a clear correlation between the amount of regulator and signal strength (for the complete dataset see supplementary Table S2 in Additional data file 1). These linear dose-response relations suggest a direct regulatory activity, while non-linear relations point at a more indirect mode of activity. Consistent with a more direct regulation on *Ath5*, 71% of the genes annotated as nucleic acid binding showed a linear dose-response in these assays (Table S2 in Additional data file 1).

To test the direct binding of some of the regulators to the promoter, namely the *bona fide *transcription factors Islet1 and p65, we screened the *Ath5 *3-kb fragment for predicted transcription factor binding sites (TFBSs) using TRANSFAC [44]. Those TFBSs located within conserved boxes proximal to the *Ath5 *transcription start site were cloned upstream of a luciferase reporter (Figure S4a in Additional data file 1). Fragments (29 bp including the TFBSs) were then assayed for their ability to mediate either Islet-1 or p65-induced transcription in a dose-response manner. Our *in vitro *analysis showed that selected TFBSs are functional by themselves (Figure S4b in Additional data file 1), thus suggesting that some of the identified regulators have a direct input on the *Ath5 *promoter.

The list of genes with a linear dose-response curve also contains enzymes, such as GPI deacetylase or thiolase, and signaling components, such as Idax and sFRP1, whose functions suggest a more upstream entry into the Ath5 regulatory pathway. In addition, several genes with unknown function showed dose-dependent behavior in our assays. To test whether the linear dose-response of these candidates with unknown function correlates with nuclear localization, we generated carboxy-terminal green fluorescent protein (GFP)-tagged proteins and analyzed their subcellular localization. Fusion constructs were co-transfected into BHK21 cells together with a red fluorescent protein membrane marker as a reference. The splicing factor SRP40 was used as a control for nuclear localization (Figure 4c). Our analysis showed that the zinc finger protein 161 and the ELG-protein are exclusively localized in the nucleus (Figure 4p, q). The RNA-binding protein RBPMS2 and Ndrg3 are localized in both the nucleus and cytoplasm (Figure 4n, o), suggesting that they can shuttle between these cellular compartments. In fact, nuclear localization of Ndrg3 has been recently reported in the mouse central nervous system [45]. The NHL-domain protein is excluded from the nucleus and accumulates in a perinuclear compartment, which resembles the Golgi apparatus (Figure 4r). In conclusion, the nuclear localization of four out of five uncharacterized proteins analyzed suggests that they act as direct regulators of *Ath5*.

### Clonal analysis of individual regulators in transgenic medaka embryos *in vivo *validates their role in RGC differentiation

**Figure 5:**
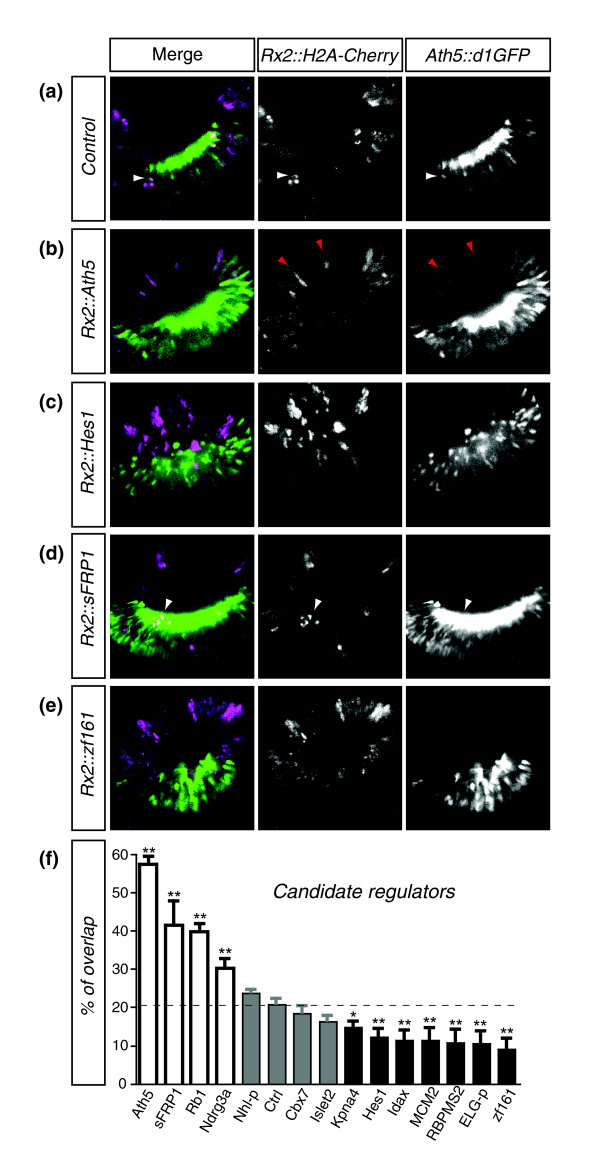
Targeted overexpression analysis. **(a-e) **Reporter expression. Optical confocal sections through stage 26 retina of *Ath5::d1GFP *transgenic medaka at the level of the lens. Embryos were co-injected with *Rx2::candidate *and *Rx2::nuclearCherry *at the one-cell stage. White arrowheads indicate representative double-labeled cells. Red arrowheads indicate the ectopic differentiation of *Ath5*-positive neurons in the peripheral retina upon *Ath5 *over-expression **(f) **Analysis of reporter overlap. For each candidate the percentage of overlap between the regulator and *Ath5*-positive cells is plotted, with error bars indicating the standard error. The significance of the differences was explored by one-way Anova analysis followed by Dunnett's post-tests to compare each value with the control. Values significantly higher (*P *< 0.01) than the control are shown by white bars, and percentages significantly lower by black bars. Percentages that deviate non-significantly from the control are shown by grey bars.

We complemented the characterization of candidate regulators by testing *in vivo *the activity of members of each of the four expression-activity categories in medaka embryos. To examine RGC differentiation, we followed the dynamic regulation of the *Ath5 *promoter using a transgenic line expressing degradable GFP under the control of the *Ath5 *promoter (*Ath5::d1GFP*). Candidates were expressed in the developing neural retina in a mosaic fashion by DNA microinjection of the candidate genes under the control of the retina-specific medaka *Rx2 *promoter. Clones expressing the candidate genes were traced by co-injection of *Rx2::H2A-mCherry *[46]. In this mosaic situation we quantified the proportion of candidate expressing cells (red) that regulated the expression of the *Ath5 *reporter (green), making the analysis independent of the total number of Ath5 positive cells. We thus determined the *in vivo *activity of the different candidates in the generation of *Ath5 *positive cells and, hence, in RGC neurogenesis. As a baseline control, the *Rx2::nuclearCherry *construct was injected alone (Figure 5a). In this assay the known regulators Ath5 and Hes-1 resulted in robust activation and repression of the reporter (Figure 5b, c). In agreement with the reported key role of Ath5 in RGC neurogenesis, its clonal expression was sufficient by itself to induce ectopic differentiation foci in the peripheral retina (Figure 5c).

Consistent with their behavior in our transactivation screen, sFRP1, Rb1 and Ndrg3a act as activators of *Ath5 in vivo *(Figure 5d, f). Interestingly, although the over-expression of these activators enhanced *Ath5 *expression, ectopic differentiation foci were never observed, suggesting that alone they do not act as instructive factors for RGC differentiation. Likewise, the candidate repressors KPNA4, MCM2, Idax, RBPMS2, ELG and Zfp161 (Figure 5e, f) down-regulated *Ath5 in vivo *and inhibited neurogenesis. Three of the candidates tested, NHL-protein, Cbx7 and Islet-2, did not significantly alter reporter expression, although they exhibited a clear effect in the screen and the dose-response analysis (Figure 5f). Taken together, 75% of the candidates tested clearly regulate *Ath5 *expression *in vivo *and activate or repress *Ath5 *as predicted from the *in vitro *assays.

Here, we present a comprehensive TRS with a detailed analysis of candidate expression patterns relative to *Ath5 *during the neurogenic wave. We analyze the subcellular localization of previously uncharacterized candidates and show that identified proteins regulate RGC neurogenesis *in vivo *in the medaka retina. Our data highlight the power of the technology to obtain an enriched set of true-positive regulators from an unbiased collection of full-length cDNAs.

## Discussion

The identification of the components of GRNs is essential to understand how specific developmental programs are executed during embryogenesis [47]. An increasing number of regulatory interactions have been already identified through microarray analysis, chromatin immunoprecipitation-based methodologies and bioinformatics approaches. While these technologies can be applied to systematically detect gene batteries downstream of a given component of the network and are powerful tools to detect individual upstream inputs, they have limited use as genome-wide screening technologies upstream of a node.

We developed the TRS approach to identify upstream factors from a large collection of cDNAs in an unbiased manner. We focused on genes acting in the control of *Ath5*-mediated RGC specification.

### The transregulation screen robustly identifies high-confidence candidate regulators of *Ath5*

The TRS approach represents a high-throughput method to explore the transregulatory activity of transcriptome-scale sets of genes acting on native promoter sequences. Using a rapid screening procedure and taking advantage of a high-quality medaka unigene expression library, our procedure overcomes many of the limitations of previously used approaches. We benchmarked the assay setup using the known *Ath5 *regulators Hes-1, Pax6 and Ath5 itself, confirming that Hes1 represses the *Ath5 *promoter, while Pax6 and Ath5 activate the promoter under screening conditions. We have screened 44% of the protein-coding genes predicted in medaka (Ensembl 50) for their activity on the *Ath5 *promoter. The internal control provided by a dual luciferase-based approach allows efficient data normalization. Due to the basal activity of the native promoter, both putative repressors and activators are identified in a single screen.

The TRS intrinsically favors the identification of direct regulators whose input on *Ath5 *may be mediated either by direct binding to the promoter or through their participation as cofactors in transcriptional complexes. Accordingly, the collection of candidate genes identified is enriched in transcriptional regulators: 44.3% of candidates are annotated as nucleic acid binding proteins (22% as transcription factors), while the percentage of transcription factors in vertebrate genomes is estimated at between 5 and 10% [48]. In fact, most of the previously uncharacterized candidates show nuclear localization. By screening the activity of medaka proteins on a medaka promoter in the context of a mammalian cell line, only strongly conserved interactions were picked up by the screen. Notably, the screen was not restricted to direct regulators only, and also identified a number of upstream regulators, such as sFRP1, a secreted molecule that has been shown to promote RGC differentiation in chicken [23].

To limit the number of selected genes to high-confidence candidates, we applied very stringent selection criteria (Figure 2b), accepting a number of false-negatives. In general, the rate of false negatives obtained by TRS will depend critically on the quality and coverage of the reference cDNA library as well as on the DNA preparations employed during the procedure. In our analysis we further discriminated false-positives by employing a pipeline of two nested screens, firstly collecting luciferase data and secondly obtaining spatio-temporal information about the candidate genes in the *in situ *hybridization screen. Indeed, we identified a number of transcription factors that clearly regulate the promoter but are expressed elsewhere in the embryo. Interestingly, each of these TFs belonged to a protein family out of which at least one member was found to be expressed in the eye. The expression pattern filter efficiently removed these false entries and the number of candidates was thus reduced from 93 to 41 genes with a spatio-temporal expression specific to subdomains of the eye.

To assess the relevance of the findings in the context of an embryo, we carried out functional assays *in vivo *and found that 9 out of 12 genes tested by clonal analysis in the developing medaka retina regulate *Ath5*. This *in vivo *validation demonstrates the power of the approach to efficiently identify high-confidence targets. We classified our candidate regulators into four different groups according to expression pattern and regulatory activity:

### Four spatially and functionally distinct sets of regulators define different phases of *Ath5 *expression

#### Activation phase

##### Group 1 genes

Group 1 genes repress the *Ath5 *promoter to prevent premature neurogenesis. Genes for MCM2, a member of the general replication initiation complex, and an importin, KPNA4, are expressed with *Ath5 *only prior to exit from the cell cycle in the most apical cells and repress the *Ath5 *promoter in the context of the retina (Figure 5b), maintaining the RPCs in an undifferentiated state. Interestingly, *MCM2 *downregulation has been correlated with cell cycle exit and Rb hyperphosphorylation [49].

##### Group 2 genes

Group 2 genes act as initial activators and are expressed in RPCs and nascent RGCs. We demonstrate that the cell cycle exit regulator Rb activates the *Ath5 *promoter *in vivo*. The onset of Rb overlaps with the last mitosis in apically located RPCs at the onset of *Ath5 *expression. A link between cell cycle exit and RGC specification has long been proposed [27] and loss of Rb has been reported to cause neuronal differentiation defects [50]. Our findings further indicate that the Rb pathway molecularly links cell-cycle exit and activation of the proneural gene *Ath5*. Interestingly, in *Drosophila *eye imaginal discs, increased *Rb *expression flanking the *atonal *domain in the furrow has been reported [51], indicating that part of the *atonal*/*Ath5 *gene network is evolutionarily conserved.

We also show that the Wnt-signal modulator sFRP1 activates *Ath5 in vitro *and *in vivo*. This substantiates and extends a previous report showing that *sFRP1 *favors generation of RGCs and photoreceptors in the developing chicken retina [23]. These findings indicate that transregulatory input from both cell-intrinsic factors and extracellular signals converges on the *Ath5 *promoter to precisely control the timing of RGC differentiation.

#### Downregulation phase

##### Group 3 genes

Group 3 genes maintain *Ath5 *expression in basal RGCs just prior to the downregulation of *Ath5*. Several reports indicate that *Ath5 *expression is maintained through an auto-regulatory feedback loop in migrating RGCs [30,33]. We hypothesize that late activators, such as Islet-2 and Ndrg3, cooperate with this feedback loop to maintain high levels of *Ath5 *mRNA until the sharp downregulation prior to RGC terminal differentiation. In agreement with this, it has been shown that *islet-2 *is a downstream target of Ath5 in mice [52].

##### Group 4 genes

The last step of *Ath5 *regulation requires sharp downregulation of the promoter. This repression requires both breaking the auto-regulatory feedback loop and directly repressing the regulatory elements of the promoter. We identified eight repressors that are expressed in late RGCs and overlap with *Ath5 *only in very few basal cells. RBPMS2, an RNA binding protein, and a chaperone-like ELG-protein [53] repress *Ath5 in vivo *(Figure 5b). We propose that they are required to break the positive *Ath5 *feedback loop on the level of RNA regulation. It is likely that the zinc finger protein Zfp-161 represents a more direct transcriptional regulator of *Ath5*, based on its protein structure, its nuclear localization, and the clear dose-response. We demonstrate that the Wnt-regulator Idax represses *Ath5 in vivo*, consistent with the notion that repression of Wnt signaling is required for the progression of neurogenesis [54].

## Conclusions

**Figure 6:**
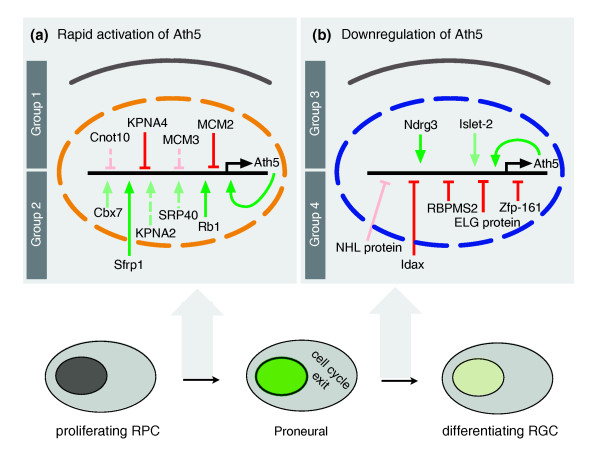
Model of *Ath5 *expression dynamics. Summary of input on the 3-kb *Ath5 *promoter fragment. Solid lines represent activities that were confirmed *in vivo*, and shaded solid lines represent regulatory activities that were not significant *in vivo*. **(a) **Onset of *Ath5 *expression. Group 1 genes such as *MCM2 *and *KPNA4 *may act to prevent premature *Ath5 *expression. *Ath5 *expression is switched on by group 2 genes, such as *sFRP1 *and *Rb*. **(b) **Downregulation of *Ath5*. Group 3 genes: Ndrg3 and Islet-2 may prevent premature downregulation of *Ath5*. Expression of *Ath5 *is downregulated by signals through Idax and the regulatory activity of RBPMS2, ELG-protein and Zfp-161 (group 4).

Our analysis establishes a regulatory framework of RGC neurogenesis. We have identified novel repressors in proliferating RPCs in addition to the previously described initial repressor Hes1 (Figure 6a). We propose that lifting of repression is accompanied by a rapid activation of the promoter through cell-extrinsic (for example, sFRP1) and cell-intrinsic (for example, Rb) inputs. The main phase of strong expression has been previously described as being driven by the *Ath5 *autoregulatory feedback loop and by NeuroM activity. We have now identified other factors that act during this phase and contribute to sustaining the *Ath5 *regulatory loop. Importantly, we describe a number of genes involved in the final downregulation of *Ath5 *(Figure 6b). These include not only transcription factors, but also regulators of RNA metabolism, enzymes and genes of as yet uncharacterized molecular function.

Our nested screening approach has been highly efficient at identifying relevant inputs to *Ath5*, a central node in the retinal neurogenic GRN. The TRS technology extends the current model of *Ath5 *regulation and gives novel insights into the mechanisms of retinal neurogenesis.

## Materials and methods

### Medaka stocks

The Cab-strain of wild-type medaka (*Oryzias latipes*) were kept in closed stocks at the EMBL, as described [55]. Embryonic stages are according to [56]. For stages after the onset of eye pigmentation the Heino strain was used [57].

### Unigene full-length library and reporter vector

Total RNA was extracted from medaka stages 18, 24, 32 and adults. mRNA was isolated using polyT-beads. The normalized full-length cDNA library was prepared using standard reverse transcription. The cDNAs were cloned into pCMV-Sport6.1 vector. We sequenced and clustered 55,296 clones based on sequence alignment. One bacterial clone of each cluster was transferred into a consolidated library in a 384-well plate by the in-house genomics service. In addition to 13,837 clones from clusters in the initial library, 3,689 clones not sequenced successfully were included.

### Reporter vector

A 3-kb fragment upstream of the medaka *Ath5 *gene (ENSORLG00000013722) was amplified using specific primers (forward primer, TGCATCTTCAGCGCAGTGGCA; reverse primer, GGTTTCTGTGCAAAGAGGCGAA) and cloned into the pGL3 luciferase reporter vector that contains the *Photinus pyralis *luciferase gene.

### Cell culture and transfection

Syrian Hamster Fibroblast (BHK21) cells were cultivated in Dulbecco's modified Eagle's medium (DMEM), supplemented with 10% fetal calf serum, 200 U/l penicillin, 200 μg/l streptomycin and 2 mM L-glutamine. Cells were incubated at 37°C in a humidified atmosphere with 5% CO_2_. For luciferase assays cells were seeded in white 96-well plates and grown for 5 h. pGL3 *Ath5::luc *vector (40 ng) and pRL-CMV control vector (5 ng) (Promega, Mannheim, Germany) were incubated with 2 μl (10 to 300 ng) of pCMV-Sport6.1::cDNA in a 3- to 6-fold volume-excess of FuGENE6 transfection reagent in serum-free medium for 30 minutes and then added to the cells. After 42 h growth at 37°C, the medium was removed and cells were lysed with 20 μl of 1× passive lysis buffer (Promega). Each clone was assayed in triplicate.

Dose-response behavior of the candidate regulators was assessed in triplicate by co-transfecting 20, 40, 80 or 160 ng of pCMV-Sport6.1::cDNA with the reporter and control construct. The total amount of DNA transfected in each dose-response experiment was kept constant by adding pCS2+ vector where appropriate (see supplementary methods in Additional data file 1).

### Luciferase assays

Raw luminescence values were sequentially recorded in a Victor Light Luminescence counter (PerkinElmer Waltham, Massachusetts, USA) using a dual flash luciferase system (Promega) according to manufacturer's guidelines. All values were stored in a FileMaker database (FileMaker, Inc, Santa Clara, California, USA) The median of the raw ratio between the firefly luciferase and the *Renilla *luciferase of each triplicate was normalized against the median of all ratios of the 96-well plates containing the triplicates. Clones with a non-random deviation from the average were selected as candidate regulators (see supplementary methods in Additional data file 1). When only one assay was available for a clone, no standard deviation filter was applied.

### Riboprobe preparation

The insert with a 3' flanking T7-promoter was amplified from the pCMV-Sport6.1 backbone using standard PCR with M13frw and M13rev primers. T7 RNA polymerase-based transcription was performed as previously described [58].

### Robotic-assisted whole-mount *in situ *hybridization

Fixation, protein K digestion and post-fixation of embryos were carried out as previously described [58]. Solutions were prepared according to standard protocols, except phosphate-buffered saline-Tween 20 (PTW) for the washes containing 0.35% PTW. In addition to the standard protocol, a pre-staining buffer (0.1 M Tris pH7.5, 25 mM NaCl, 0.1% Tween20) was prepared. Hybridization and washes were carried out in an *in situ *robot (InSitu Pro, Intavis Koeln, Germany) with an adapted *in situ *protocol (see supplementary Materials and methods in Additional data file 1). The staining reactions were performed manually as previously described [58].

### Double-fluorescence *in situ *hybridization

Embryos were prepared as described above for single-color *in situ *hybridization. Fluorescein-labeled probes were generated against *Ath5 *according to standard protocol [59]. Embryos were hybridized with a mixture of this probe and a DIG-labeled probe against the candidate regulator. Anti-fluorescein antibody (α-Fluo Ab) coupled to peroxidase and anti-digoxigenin antibody (α-Dig Ab) coupled to alkaline phosphatase were mixed in blocking buffer at 1:500 and 1:1,000 dilutions, respectively. Embryos were first incubated with TSA-fluorescein according to the manufacturer's instructions (PerkinElmer) for 60 minutes at room temperature in the dark. The DIG-probes were visualized with FastRed staining (Roche Mannheim, Germany).

### Imaging

Images from embryos mounted *in toto *were acquired on a Leica SP2 confocal microscope using a HCX PL APO CS 40×, 1.25 oil objective. Images were assembled and processed in Adobe Photoshop and ImageJ. Brightness and contrast were adjusted; filtering was applied for noise reduction and thresholding when needed.

### Conservation analysis and transcription factor binding site discovery

Conserved sequences in the 3-kb upstream sequence of *Ath5 *were determined as previously described [31]. Position weight matrices for the TFBSs were obtained from TRANSFAC [44] (NFkB Identifier: M0019); for islet-2 we used the position weight matrix for the LIM- and homeodomain-containing transcription factor Lhx3 (M00510). Potential TFBSs were search for using Possum [60], using a threshold of 4 and a relative abundance range of 50 for M00194 and 100 for M00510.

### Fusion protein and localization assays

The proteins were amplified from pCMV-Sport6.1 using the specific primers (see supplementary Materials and methods in Additional data file 1). Forward primers contained restriction sites creating an *Eco*RI compatible overhang and the reverse primers a restriction site creating an *Nco*I compatible overhang. The sequence verified fragments were subcloned into a pCS2+ hGFP vector.

BHK21 cells were grown on human fibronectin-coated culture slides to 40 to 50% confluency and transfected with 0.8 μg of each fusion protein and 0.2 μg of pCS2+ lynd-tomato for membrane labeling. After 42 h cells were fixed in 4% paraformaldehyde/phosphate-buffered saline, permeabilized in 0.2%Triton/phosphate-buffered saline and stained with DAPI. The slides were sealed with a glass coverslip and were kept at 4°C in the dark.

### Functional assays

Candidate regulators in pCMV-Sport6.1 were cloned into a gateway recombination destination vector containing a 2.4-kb *OlRx2 *promoter fragment driving expression in the eye [61]. Each construct (5 ng/μl) was individually co-injected with *Rx2::H2A-Cherry *(7.5 ng/μl) into one-cell stage medaka embryos as previously described [46]. Stage 26 embryos were protein K treated to remove hairs from the chorion, heptanol treated (3 mM) and imaged *in vivo *using a Leica SPE confocal microscope with a 40× dipping lens. Retina with clonal expression of the constructs were imaged individually at the level of the lens. H2A-Cherry-expressing cells within the *Ath5*-expression domain were scored for *Ath5 *coexpression.

### Statistical analysis

Quantitative data are expressed as mean ± standard error of the mean. Significant differences among groups were evaluated by one-way ANOVA followed by Dunnett's tests (GraphPad Prism. GraphPad Software, Inc. San Diego, California, USA) and are indicated when relevant.

## Abbreviations

bHLH: basic helix-loop-helix; GFP: green fluorescent protein; GRN: gene regulatory network; IS: initiation stage; PS: progression stage; Rb: Retinoblastoma; RGC: retinal ganglion cell; RPC: retinal progenitor cell; sFRP: secreted frizzled related protein; SWS: steady wave stage; TFBS: transcription factor binding site; TRS: trans-regulation screen.

## Authors' contributions

JRMM and JW conceived the study. JRMM and MS designed, coordinated and carried out the screening pipeline. MS designed and maintained the screen database. BW carried out the semi-automated *in situ *hybridizations. PM participated in the double fluorescence whole-mount *in situ *hybridization experiments. The manuscript was initially drafted by MS. JRMM and JW worked on further versions of the text. All authors read and approved the final manuscript.

## Additional data files

The following additional data are available with the online version of this paper: a PDF including supplementary Tables S1 and Table S2, supplementary Figures S1 to S4, and supplementary Materials and methods (Additional data file 1).

## Supplementary Material

Additional data file 1 Table S1: a complete candidate list. Table S2: values obtained in dose-response experiments for the different candidates. Figure S1: behavior of known regulators under screening conditions. Figure S2: representative pictures form the automated *in situ *hybridization screen. Figure S3: double-fluorescent whole-mount *in situ *hybridization of additional candidates. Figure S4: functional analysis of TFBSs within the *Ath5 *3-kb regulatory sequence. Supplementary Materials and methods provide a detailed description of the transregulation and *in situ *hybridization screens. Click here for file
