# Supplementary material for: A global survey identifies novel upstream components of the Ath5 neurogenic network
Source: Genome Biol. 2009 Sep 7;10(9):R92. doi: 10.1186/gb-2009-10-9-r92 (PMC2768981; doi:10.1186/gb-2009-10-9-r92)
Supplement: Additional data file 1 — Table S1: a complete candidate list. Table S2: values obtained in dose-response experiments for the different candidates. Figure S1: behavior of known regulators under screening conditions. Figure S2: representative pictures form the automated in situ hybridization screen. Figure S3: double-fluorescent whole-mount in situ hybridization of additional candidates. Figure S4: functional analysis of TFBSs within the Ath5 3-kb regulatory sequence. Supplementary Materials and methods provide a detailed description of the transregulation and in situ hybridization screens. [file gb-2009-10-9-r92-S1.pdf]

Supplemental Online Material

A global survey identifies novel upstream components of the *Ath5* neurogenic network

Marcel Souren, Juan Ramon Martinez-Morales, Panagiota Makri, Beate Wittbrodt, Joachim Wittbrodt

Supplemental material includes 2 supplementary tables, 4 supplementary figures and supplementary methods.

Figure legends

Table S1. Complete candidate list

For each clone the fold-change of reporter activity is shown. For each individual gene, expression patterns were determined at all three stages of the wave spread using standard WISH. Wherever was possible, ensembl IDs were assigned to the clones, otherwise we assigned identifiers corresponding to the medaka genome project.

Table S2. Dose response experiments

(A) Dose-response data using CMV promoter-driven control reporter (B) Dose-response data using SV40 promoter-driven control reporter For each clone four different concentrations of candidate regulator were transfected. Candidates showing a linear dose-response are indicated in both.

Figure S1. Behavior of known regulators under screening conditions

Known regulators of Ath5 were added at increasing concentration to the reporter assay. X-axis denotes the amount of regulator added per well. The y-axis shows fold-changes of luminescence normalized against an empty vector control. Ath5

and Pax6 effect as activators, and Hes-1 repressive activity are consistent with previously published results.

Figure S2. Automatized in situ hybridization screen.

Representative examples of expression patterns obtained in *in situ* hybridization screen. Expression was determined at three stages, initiation stage (stg24), progression stage (stg27) and steady wave stage (stg31). Relevant stages for representative examples are shown as dorsal whole mount images, anterior is to the left. A-F Group 1, repressors expressed in RPCs; G-I Group 2, SRP40, an activator expressed in RPCs; J,K Group 3, Islet-2, activator expressed in RGCs, L, RBPMS2, repressor in RGCs. M: ubiquitously expressed gene, p65; N,O genes expressed elsewhere, cytokeratin and MBX-S.

Figure S3. Double-fluorescent whole-mount in situ hybridization of candidates (supplementary)

All images are single horizontal confocal sections of the developing eye at the level of the lens with the embryo facing left. *Ath5* mRNA was detected using TSA-fluorescein (shown in green), regulator mRNA was visualized using FastRed staining (shown in purple).

Figure S4 Analysis of 3kb regulatory Ath5 sequence

(A) conservation and TFBS analysis. Conserved sequences within fish species are shown in green. Putative transcription factor bindings sites (TFBS) were detected using the respective PWMs.

(B) Functional test of conserved TFBS. For each of the conserved TFBS labeled in red in (A) a 24bp fragment containing the TFBS was cloned upstream a luciferase gene coupled to a minimal promoter. The reporters were transfected with increasing amounts of the respective regulator under the control of the CMV-promoter. The relative luminescence normalized against a pCS2+ control is represented in the graphs. The numbers in brackets refer to the numbers TFBS in (A)

Supplementary table 2 – dose-response data

(A)

Name	GO	20ng/ well	40ng/ well	80ng/ well	160ng/ well
**Clones with a linear dose-response**					
ARG1		1.45 ± 0.06	0.48 ± 0.03	0.61 ± 0.03	0.61 ± 0.01
Bcat2	Nucleic acid binding	0.97 ± 0.05	1.11 ± 0.02	1.88 ± 0.21	1.77 ± 0.12
cbx7	Nucleic acid binding	0.37 ± 0.05	1.97 ± 0.12	2.88 ± 0.04	4.18 ± 0.14
CEB55		0.84 ± 0.11	1.06 ± 0.09	1.08 ± 0.04	1.62 ± 0.32
Cnot10	Other transcription factor	0.48 ± 0.07	0.50 ± 0.02	0.53 ± 0.03	0.71 ± 0.02
ELG protein		0.47 ± 0.07	1.06 ± 0.03	2.12 ± 0.51	1.98 ± 0.15
FAN		0.66 ± 0.02	0.99 ± 0.05	1.02 ± 0.03	1.27 ± 0.07
GPI deacylase	Nucleic acid binding	0.99 ± 0.09	1.24 ± 0.04	1.55 ± 0.08	2.42 ± 0.29
Idax		0.33 ± 0.04	0.34 ± 0.00	0.32 ± 0.02	0.23 ± 0.03
Islet2	Transcription factor	1.45 ± 0.10	2.27 ± 0.32	3.76 ± 0.17	7.27 ± 2.01
KPNA4	Other transcription factor	0.64 ± 0.04	0.80 ± 0.04	0.66 ± 0.03	0.58 ± 0.02
MCM3	Nucleic acid binding	0.68 ± 0.05	0.69 ± 0.07	0.64 ± 0.01	0.56 ± 0.03
Ndrg3a		0.50 ± 0.03	0.61 ± 0.07	0.74 ± 0.02	1.31 ± 0.32
NHL-protein		0.74 ± 0.16	0.47 ± 0.03	0.38 ± 0.02	0.48 ± 0.04
p65 TF	Transcription factor	0.57 ± 0.03	1.43 ± 0.07	2.13 ± 0.03	5.15 ± 1.07
PTPN2		1.06 ± 0.06	0.93 ± 0.07	1.29 ± 0.07	1.45 ± 0.01
Rb1	Other transcription factor	1.09 ± 0.08	1.04 ± 0.10	1.44 ± 0.07	1.71 ± 0.01
RBPMS2	Nucleic acid binding	0.61 ± 0.03	0.60 ± 0.04	0.63 ± 0.06	0.75 ± 0.05
sFRP-1		0.59 ± 0.03	0.79 ± 0.06	0.41 ± 0.08	0.39 ± 0.09
SRP40	Nucleic acid binding	0.76 ± 0.08	1.82 ± 0.11	2.28 ± 0.19	3.11 ± 0.27
Sterol demethylase		1.47 ± 0.08	1.40 ± 0.15	1.86 ± 0.07	2.56 ± 0.21
Thiolase		0.87 ± 0.10	0.97 ± 0.04	1.21 ± 0.18	2.06 ± 0.27
Transferase		1.05 ± 0.05	1.12 ± 0.11	1.36 ± 0.05	1.50 ± 0.06
USP25		0.59 ± 0.06	0.64 ± 0.10	0.72 ± 0.04	0.62 ± 0.07
Zfp 161		0.61 ± 0.02	0.40 ± 0.02	0.34 ± 0.02	0.28 ± 0.02
**Clones without a linear dose-response**					
ATP-Synthase		1.08 ± 0.05	0.72 ± 0.07	0.72 ± 0.05	8.58 ± 1.83
bub3		0.62 ± 0.02	0.74 ± 0.02	0.71 ± 0.03	1.01 ± 0.28
DuS4L		0.80 ± 0.05	1.01 ± 0.12	1.31 ± 0.06	1.95 ± 0.13
EF-1-alpha		1.03 ± 0.18	0.84 ± 0.05	1.15 ± 0.07	1.55 ± 0.14
Hsp1		0.74 ± 0.13	0.99 ± 0.09	1.02 ± 0.06	1.33 ± 0.22
KPNA2	Other transcription factor	1.08 ± 0.03	0.93 ± 0.06	1.12 ± 0.02	1.38 ± 0.04
MCM2	Other transcription factor	0.84 ± 0.04	1.12 ± 0.04	1.47 ± 0.03	1.58 ± 0.03
MRPL47	Nucleic acid binding	0.67 ± 0.05	0.83 ± 0.07	0.73 ± 0.07	0.85 ± 0.06
NHL-protein II		0.85 ± 0.04	0.75 ± 0.04	1.00 ± 0.05	1.09 ± 0.08
RBM4L		0.56 ± 0.02	0.51 ± 0.02	0.70 ± 0.15	0.60 ± 0.03
Ribonuclease	Other transcription factor	0.80 ± 0.05	0.84 ± 0.04	0.89 ± 0.06	1.21 ± 0.21
Samd11		0.79 ± 0.16	0.83 ± 0.07	0.86 ± 0.06	1.47 ± 0.23
TARBP2	Nucleic acid binding	0.61 ± 0.06	0.74 ± 0.06	0.58 ± 0.03	0.84 ± 0.06

Tetraspanin-9	Other transcription factor	0.76 ± 0.06	0.73 ± 0.01	0.93 ± 0.01	1.17 ± 0.04
TMEM79		0.64 ± 0.04	1.06 ± 0.08	1.09 ± 0.07	1.54 ± 0.06
tubulin-like		0.60 ± 0.07	0.71 ± 0.03	0.95 ± 0.02	0.89 ± 0.03
UBR2		0.80 ± 0.19	1.04 ± 0.04	1.03 ± 0.03	0.00 ± 0.00
Uncharacterized 3		1.28 ± 0.09	0.73 ± 0.03	1.01 ± 0.06	1.33 ± 0.16
USP1	Nucleic acid binding	0.57 ± 0.02	0.97 ± 0.03	0.67 ± 0.02	0.81 ± 0.03
WDR43		0.67 ± 0.11	1.43 ± 0.19	1.39 ± 0.09	1.23 ± 0.05

(B)

Name	20ng/ well	40ng/ well	80ng/ well	160ng/ well
**Clones with a linear dose-response**				
Bcat2	0.91 ± 0.07	1.04 ± 0.10	1.35 ± 0.15	1.59 ± 0.20
Cbx7	1.25 ± 0.06	1.54 ± 0.16	1.83 ± 0.14	2.96 ± 0.06
CEB55	1.23 ± 0.14	1.07 ± 0.36	1.51 ± 0.54	2.69 ± 0.17
Cnot10	0.42 ± 0.11	0.51 ± 0.07	0.43 ± 0.03	0.44 ± 0.04
DuS4L	0.51 ± 0.04	0.52 ± 0.06	0.48 ± 0.02	0.58 ± 0.01
ELG protein	0.42 ± 0.05	0.49 ± 0.04	0.43 ± 0.07	0.56 ± 0.04
GPI deacylase	1.01 ± 0.08	1.29 ± 0.06	1.75 ± 0.39	2.18 ± 0.08
Idax	0.19 ± 0.01	0.20 ± 0.02	0.16 ± 0.01	0.17 ± 0.03
Islet2	0.95 ± 0.06	1.76 ± 0.14	2.06 ± 0.06	4.36 ± 0.41
KPNA4	0.43 ± 0.01	0.42 ± 0.03	0.38 ± 0.01	0.44± 0.04
MCM2	0.55 ± 0.06	0.62 ± 0.03	0.62 ± 0.08	0.71 ± 0.07
MCM3	0.58 ± 0.03	0.537 ± 0.018	0.445 ± 0.013	0.425 ± 0.047
Ndrg3a	0.99 ± 0.15	0.63 ± 0.02	0.70 ± 0.03	0.70 ± 0.04
NHL-protein	0.55 ± 0.03	0.59 ± 0.02	0.57 ± 0.06	0.62 ± 0.09
p65 TF	1.73 ± 0.29	7.67 ± 1.24	15.66 ± 0.62	29.58 ± 2.94
PTPN2	1.45 ± 0.42	0.90 ± 0.33	0.76 ± 0.20	0.63 ± 0.07
Rb1	2.09 ± 0.24	1.87 ± 0.41	1.38 ± 0.09	1.18 ± 0.14
RBPMS2	0.62 ± 0.07	0.55 ± 0.05	0.84 ± 0.24	7.81 ± 1.23
Samd11	0.81 ± 0.22	0.60 ± 0.06	0.57 ± 0.05	0.53 ± 0.03
sFRP-1	1.92 ± 0.13	2.75 ± 0.19	4.78 ± 0.64	6.04 ± 0.84
SRP40	0.93 ± 0.01	1.00 ± 0.08	1.13 ± 0.12	1.37 ± 0.07
Sterol demethylase	0.91 ± 0.05	1.55 ± 0.12	1.47 ± 0.03	2.60 ± 0.10
TARBP2	0.52 ± 0.03	0.31 ± 0.02	0.36 ± 0.07	0.32 ± 0.04
Tetraspanin-9	0.81 ± 0.04	0.99 ± 0.15	1.01 ± 0.08	2.15 ± 0.25
Thiolase	0.91 ± 0.11	1.23 ± 0.09	1.86 ± 0.08	3.33 ± 0.21
TMP49	0.55 ± 0.01	0.59 ± 0.03	0.70 ± 0.00	1.18 ± 0.08
USP1	0.71 ± 0.06	0.73 ± 0.16	0.38 ± 0.09	0.48 ± 0.04
Zfp 161	0.68 ± 0.04	0.63 ± 0.01	0.65 ± 0.04	0.64 ± 0.02
**Clones without a linear dose-response**				
ARG1	0.68 ± 0.10	1.11 ± 0.52	0.90 ± 0.27	0.92 ± 0.16
Bub3	0.84 ± 0.25	1.05 ± 0.29	0.79 ± 0.06	1.09 ± 0.08
KPNA2	0.88 ± 0.13	1.14 ± 0.27	0.92 ± 0.12	1.12 ± 0.12
MRPL47	0.72 ± 0.11	0.77 ± 0.03	0.62 ± 0.12	1.38 ± 0.20
Ribonuclease	0.75 ± 0.09	0.62 ± 0.03	0.76 ± 0.11	0.73 ± 0.05
Uncharacterized 3	0.77 ± 0.29	0.97 ± 0.13	0.77 ± 0.14	0.74 ± 0.16

USP25	0.85 ± 0.13	1.10 ± 0.27	0.72 ± 0.22	0.75 ± 0.06
WDR43	0.70 ± 0.05	1.17 ± 0.26	0.80 ± 0.19	0.824± 0.15

Table S1 - List of candidates

Repressors

Name	Fold-change	expressed	ID
CRTAP	0.16 ± 0.01	Elsewhere	ENSORLG00000011852
Idax	0.16 ± 0.01	In RGCs	ENSORLG00000006474
Cnot10	0.17 ± 0.02	In RPCs	ENSORLG00000003576
LPP3-like	0.18 ± 0.00	Elsewhere	ENSORLG00000006759
TOR1AIP1	0.19 ± 0.00	No expression	ENSORLG00000016657
RBPMS2	0.20 ± 0.06	In RGCs	ENSORLG00000001493
AATF	0.21 ± 0.02	In RPCs	ENSORLG00000013628
Uncharacterized 1	0.22 ± 0.01	Ubiquitous	UTOLAPRE05100106330
RBM4L	0.23 ± 0.01	In RGCs	ENSORLG00000011589
Zfp 410	0.24 ± 0.03	Elsewhere	ENSORLG00000009376
Zfp 161	0.24 ± 0.02	In RGCs	ENSORLG00000004128
KPNA4	0.25 ± 0.04	In RPCs	ENSORLG00000006226
Ikaros	0.25 ± 0.03	Elsewhere	ENSORLG00000020562
NHL-protein	0.27 ± 0.00	In RGCs	ENSORLG00000014595
MCM2	0.27 ± 0.00	In RPCs	ENSORLG00000003191
Beta-actin	0.27 ± 0.03	Ubiquitously	ENSORLG00000013676
ARG1	0.27 ± 0.02	In RPCs	UTOLAPRE05100101036
ELG protein	0.27 ± 0.00	In RGCs	ENSORLG00000013703
Tubulin-like	0.27 ± 0.04	Elsewhere	ENSORLG00000016464
Fibronectin-like	0.27 ± 0.03	No expression	ENSORLG00000002976
Actin-like	0.27 ± 0.02	Elsewhere	ENSORLG00000017368
Apo-AI	0.28 ± 0.03	Elsewhere	ENSORLG00000015744
DuS4L	0.28 ± 0.01	In RPCs	ENSORLG00000008306
USP25	0.28 ± 0.05	In RPCs	ENSORLG00000000577
MBX-S	0.28 ± 0.01	Elsewhere	ENSORLG00000008964
Hsbp1	0.28 ± 0.02	Elsewhere	ENSORLG00000015789
ATP-Synthase	0.28 ± 0.04	In RPCs	ENSORLG00000003517
WDR43	0.29 ± 0.04	In RPCs	ENSORLG00000018236

Activators

Name	Fold-change	expressed	ID
SRP40	2.88 ± 0.10	In RPCs	ENSORLG00000009268
Heatr5B	2.91 ± 0.31	No expression	ENSORLG00000012398
HMG	2.93 ± 0.49	Ubiquitously	ENSORLG00000008779
Bcat2	2.93 ± 0.27	In RPCs	ENSORLG00000001573
Cbx7	2.96 ± 0.14	In RPCs	ENSORLG00000013869
sFRP-1	2.97 ± 0.55	In RPCs	ENSORLG00000016179
Nfkbia	2.99 ± 0.44	Ubiquitously	ENSORLG00000017687
Tetraspanin 31	3.00 ± 0.38	In RGCs	ENSORLG00000016592
MCM3	3.00 ± 0.00	In RPCs	ENSORLG00000016187
FBXL3	3.00 ± 0.48	No expression	ENSORLG00000011408
TMEM79	3.01 ± 0.30	In RPCs	ENSORLG00000008212
TARBP2	3.05 ± 0.18	In RPCs	ENSORLG00000005209
GNS	3.12 ± 0.27	Elsewhere	ENSORLG00000016835
PFKFB4	3.12 ± 0.36	No expression	ENSORLG00000013355
UBR2	3.18 ± 0.36	Ubiquitously	ENSORLG00000003350
MNAT1	3.22 ± 0.00	No expression	ENSORLG00000015749
USP1	3.24 ± 0.34	In RPCs	ENSORLG00000010503
Coiled-coil contain.	3.25 ± 0.43	Ubiquitously	UTOLAPRE05100105142
PYGL	3.27 ± 0.50	elsewhere	ENSORLG00000008955
Tubulin beta-chain	3.28 ± 0.59	ubiquitously	ENSORLG00000016464
FAN	3.28 ± 0.28	in RPCs	ENSORLG00000005939
Tetraspanin-9	3.29 ± 0.00	in RPCs	ENSORLG00000016594
EF-1-alpha	3.31 ± 0.26	Ubiquitously	ENSORLG00000007614

Ankrd43	3.33 ± 0.41	No expression	ENSORLG00000005833
MRPL47	3.33 ± 0.00	In RPCs	ENSORLG00000005717
Sterol demethylase	3.33 ± 0.33	In RPCs	ENSORLG00000020002
Cytokeratin-8	3.33 ± 0.22	Elsewhere	UTOLAPRE05100109764
NHL-domain II	3.36 ± 0.59	In RPCs	ENSORLG00000014595
Transferase	3.39 ± 0.58	In RPCs	ENSORLG00000015041
Rb1	3.50 ± 0.45	In RPCs	ENSORLG00000008317
Aldh1a2	3.53 ± 0.00	Elsewhere	ENSORLG00000008319
OTCase	3.55 ± 0.00	Elsewhere	ENSORLG00000009056
PTPN2	3.65 ± 0.59	In RPCs	ENSORLG00000002826
KPNA2	3.65 ± 0.36	In RPCs	ENSORLG00000015192,
Synthropin	3.69 ± 0.16	No expression	UTOLAPRE05100112103
Ndrg3a	3.73 ± 0.00	In RGCs	ENSORLG00000013821
Sox-14	3.74 ± 0.33	Elsewhere	ENSORLG00000011685
MRTF-8	3.75 ± 0.44	No expression	ENSORLG00000005094
Jun	3.76 ± 0.24	No expression	ENSORLG00000017506
Nfkbie	3.80 ± 0.15	No expression	ENSORLG00000009824
FBXW5	3.86 ± 0.36	Elsewhere	ENSORLG00000007130
PPAP2C	3.86 ± 0.00	Elsewhere	ENSORLG00000014235
ERG	3.89 ± 0.12	Elsewhere	ENSORLG00000015940
TMP49	3.92 ± 0.42	No expression	ENSORLG00000012702
ARNT2	4.08 ± 0.78	Elsewhere	ENSORLG00000019479
Grou2 rTLE3	4.12 ± 0.09	Elsewhere	ENSORLG00000006700
Ribonuclease	4.15 ± 0.09	In RPCs	ENSORLG00000004042
Sfrs4	4.23 ± 0.23	No expression	ENSORLG00000009430
Aminoacylase-1	4.24 ± 0.00	No expression	ENSORLG00000002219
Rab-18	4.63 ± 0.00	Elsewhere	ENSORLG00000009609
CEB55	4.70 ± 0.39	In RPCs	ENSORLG00000005053
Bub3	4.85 ± 0.00	In RPCs	ENSORLG00000018829
Thiolase	4.98 ± 0.32	In RPCs	ENSORLG00000009609
Islet2	5.11 ± 0.22	In RGCs	ENSORLG00000012362
WDR45L	5.16 ± 0.77	No expression	ENSORLG00000012984
CRMp4	5.28 ± 0.61	Elsewhere	ENSORLG00000019028
Ankrd39	5.40 ± 0.71	Ubiquitously	ENSORLG00000014221
Samd11	5.50 ± 0.00	Elsewhere	ENSORLG00000005568
HoxA9	5.57 ± 0.29	Elsewhere	ENSORLG00000005025
Uncharacterized 2	5.90 ± 0.00	Elsewhere	ENSORLG00000001089
p65 TF	6.16 ± 0.81	Ubiquitously	ENSORLG00000011104
Uncharacterized 3	6.62 ± 0.62	No expression	ENSORLG00000017609
Hsp1	11.02 ± 1.36	In RPCs	ENSORLG00000020428
GPI deacylase	15.00 ± 0.81	In RPCs	ENSORLG00000018243
Deiodinase	16.77 ± 0.20	No expression	ENSORLG00000014548

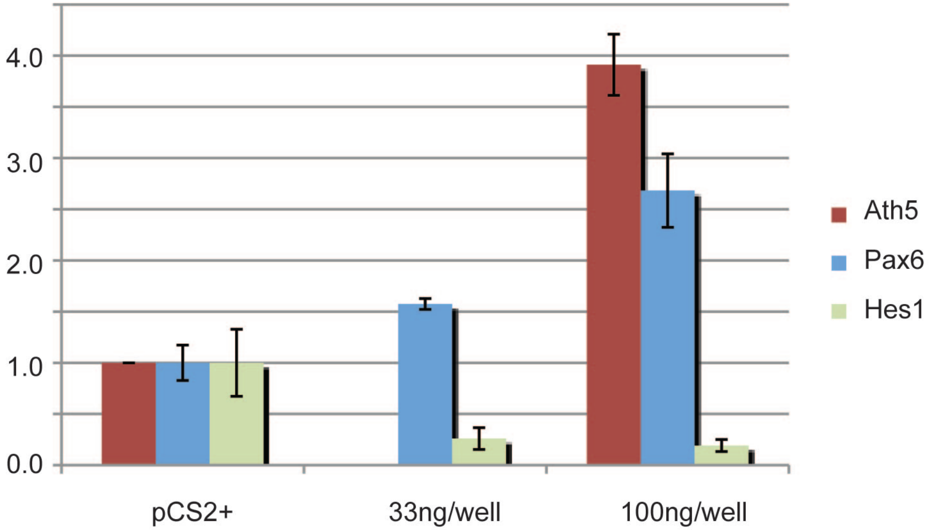

Figure S1 control regulators of Ath5

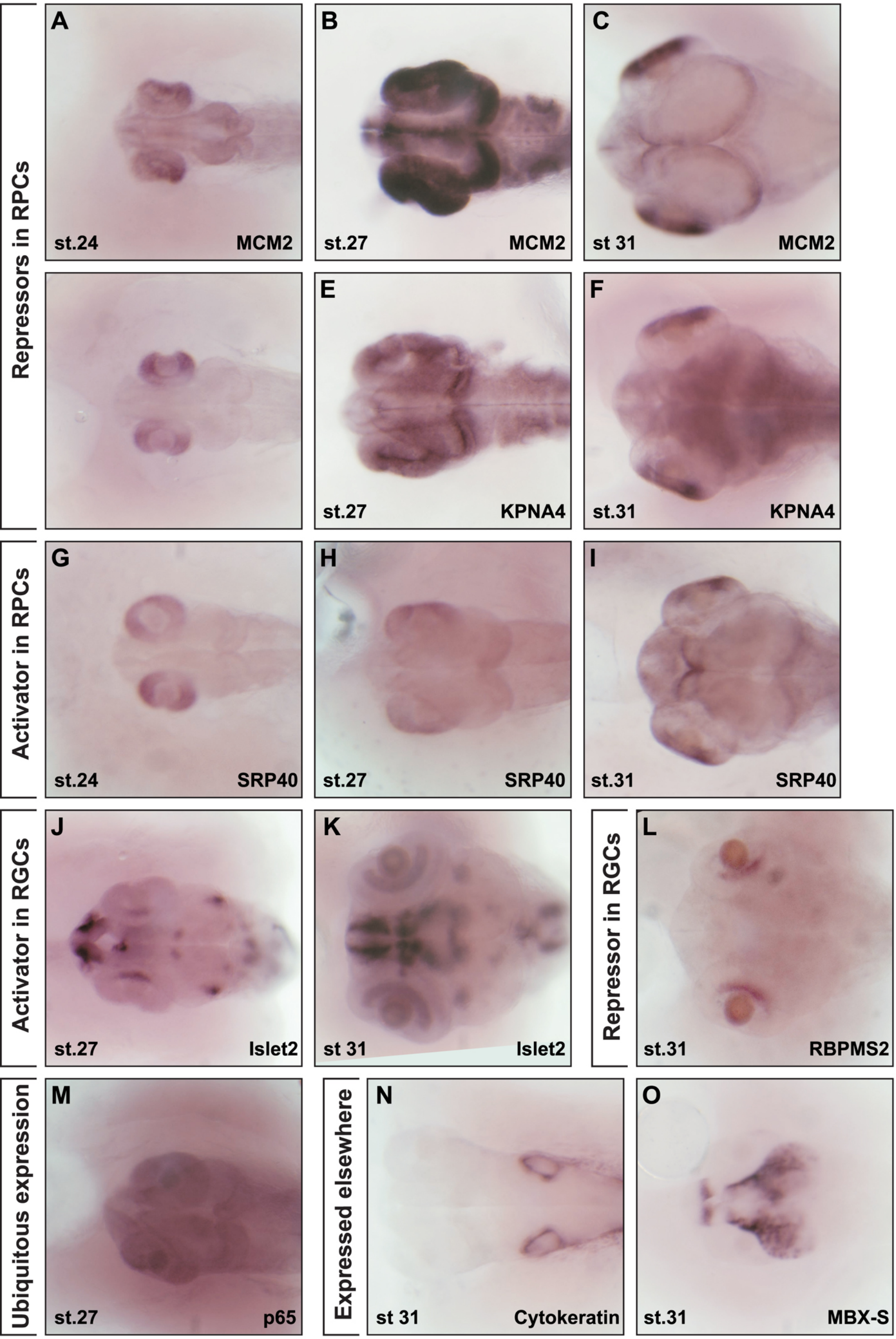

Figure S2 WISH

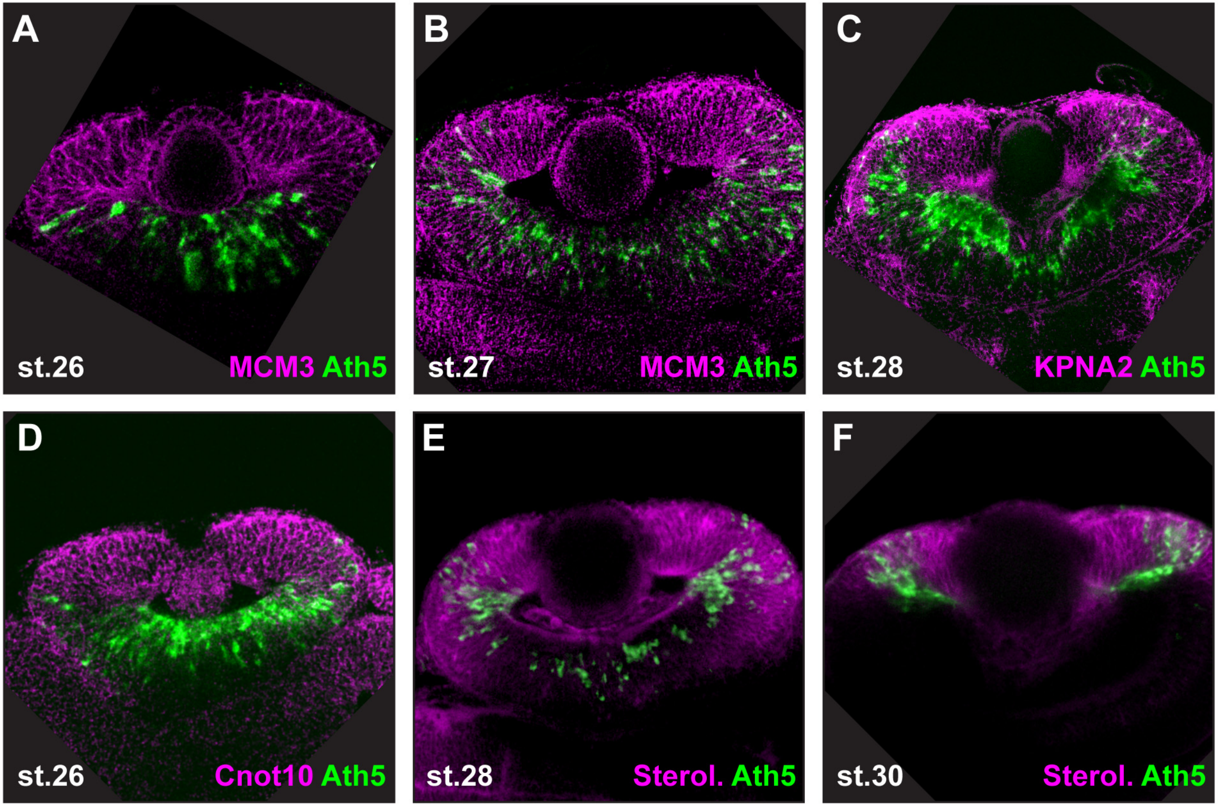

Figure S3 DWISH

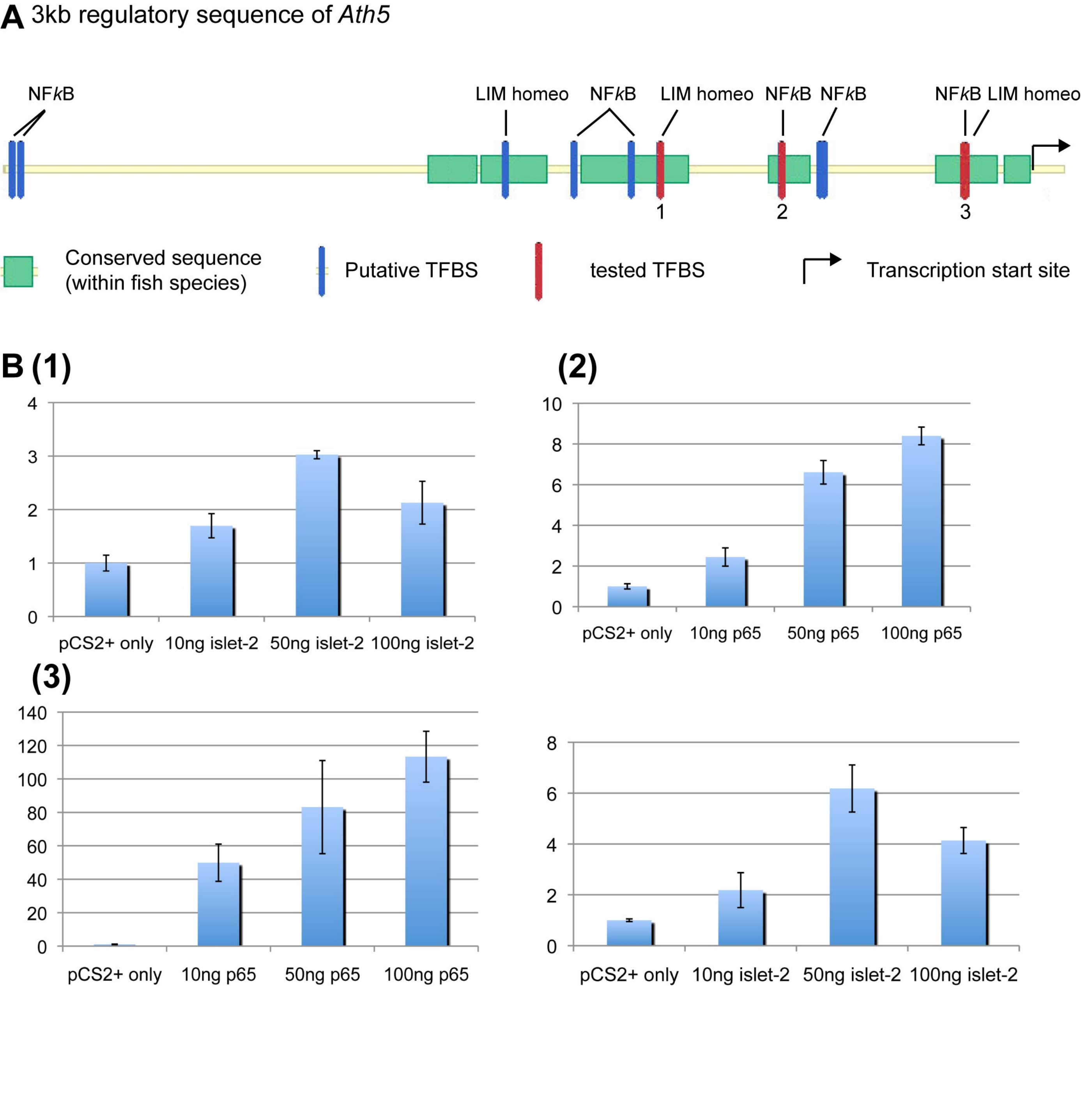
A 3kb regulatory sequence of Ath5
NFkB
LIM homeo
NFkB
LIM homeo
NFkB
NFkB
NFkB
LIM homeo
1
2
3
Conserved sequence (within fish species)
Putative TFBS
tested TFBS
Transcription start site
B (1)
4
3
2
1
0
pCS2+ only
10ng islet-2
50ng islet-2
100ng islet-2
(2)
10
8
6
4
2
0
pCS2+ only
10ng p65
50ng p65
100ng p65
(3)
140
120
100
80
60
40
20
0
pCS2+ only
10ng p65
50ng p65
100ng p65
8
6
4
2
0
pCS2+ only
10ng islet-2
50ng islet-2
100ng islet-2

Figure S4

Supplementary methods

Screening setup

For each clone the trans-regulatory effect on the *Ath5* promoter was tested in triplicate. The cDNAs were supplied as DNA mini-preparations in TE-buffer in 96-well plates by the in-house EMBL genomic service. For each round of seeding, transfection and luciferase assays 8 96-well plates of clones were assayed in triplicate, i.e. 24 96-well plates of cells were handled per round of screening. From each well 6µl containing an average of 300ng of DNA were transferred into a fresh 96-well plate using a 12-channel pipette. In parallel pGL3 *Ath5*::Luc and pRL-CMV were added to serum-free medium and mixed well. Transfection reagent was added and mixed with the DNA solution. Using an 8-channel multistep pipette 150µl of the solution were immediately added to the cDNA solution in the 96-well plates and incubated for 30min. 48µl of this DNA-transfection reagent mixture were then added to the cell as described below.

Component	Amount/ volume per well
pGL3 *Ath5*::Luc	40ng
pRL-CMV	5ng
pCMV-Sport6.1::cDNA	2µl (on average 80ng)
FuGENE6 transfection reagent	435nl

Table 1 Transfected components per well in large scale screen

Transfection and passive lysis

Prior to transfection, cells of a 9cm Petri dish at 90% confluency were trypsinised, detached from each other by pipetting up and down and resuspended in 10ml of standard medium. This stock suspension was diluted 1:30 in fresh standard medium. 200µl of this working suspension were transferred into each well of a white-bottom

96-well plate[1] using a multichannel-multistep pipette[2]. Cells were then allowed to re-attach to the bottom of the well and grow for 5h-6h until they reached 50% confluency. Prior to transfection the different plasmids were mixed with FuGENE6 transfection reagent[3] in serum-free medium[4] at room temperature. For each well 40ng of pGL3 *Ath5*::luc2 vector, 5ng of pRL-CMV vector and 10ng-300ng of pCMV-Sport6.1::cDNA were mixed with 3-6fold volume-excess of FuGENE6 transfection reagent in 50µl serum-free medium. After a 30min incubation the mixture was added drop-wise into the 96-well plates containing the cells. Cells were allowed to grow for 42h at 37°C. The medium was removed and cells were lysed with 1X passive lysis buffer[5] for 15min while shaking. To avoid loss of luciferase activity the lysate was immediately used for luminescence assays. Alternatively, plates were kept at 4°C for up to 6 hours.

Luciferase assays

Luciferase assays were carried out in a luminometer[6] containing two dispensers and a stacker device. Substrates for the assays were obtained from a commercial supplier[7]. The LARII solution was prepared in advance and frozen in aliquots at -80°C. The Stop&Glow solution was prepared freshly for each luminometer run. Leftovers were frozen for one month maximum at -80°C and mixed with fresh Stop&Glow solution. Measurements were carried out for one well after the other. After addition of 45µl LARII reagent containing the substrate luciferin for the firefly

---

[1] OptiPlate-96, White Opaque 96-well Microplate, PerkinElmer, Rodgau-Jügesheim, Germany, Cat.-No. 6005299
[2] ResearchPro, 50-1.200 µl, 8-channel, Eppendorf, Hamburg, Germany, Cat.-No. 4860 000.577
[3] FuGENE6 Transfection Reagent, Roche Diagnostics GmbH, Mannheim, Germany, Cat.-No. 11988387001
[4] Opti-MEM® I Reduced Serum Medium (1X), liquid - with L-Glutamine, Invitrogen, Karlsruhe, Germany, Cat.-No. 31985-047
[5] Passive Lysis 5X Buffer, Promega, Mannheim, Germany, Cat.-No. E1941
[6] VICTOR Light Luminescence Counter, PerkinElmer, Rodgau-Jügesheim, Germany, Cat.-No. 1420-060
[7] Dual-Luciferase® Reporter 1000 Assay System, Promega, Mannheim, Germany, Cat.-No. E1980

luciferase a delay of 1.6 seconds before starting the measurement allowed complete mixing of the reagent with the lysate. The intensity of the luminescence was recorded for 4 seconds. Subsequently 45µl of Stop&Glow solution were added through the second dispenser, quenching the luminescence of the firefly luciferase and supplying the substrate for the *Renilla* luciferase, coelenterazine. After another delay of 1.6 seconds the luminescence of the control luciferase was recorded for 2 seconds. See complete program in supplementary methods.

Dose-response assays

Amount of candidate cDNA	Amount of pCS2+
12.5ng	87.5ng
25ng	75ng
50ng	50ng
100ng	0ng

Table 2 Dose-response candidate cDNA amount per well

Dual-luciferase assay program for Victor Light luminometer

	Description	Specifications	Values
1	Injection of LARII solution	Injector	1
		Speed	5
		Volume	45 µL
		Increment	0 µL
		Replicate	1
		Injection mode	aspVol=dispVol
		Repeated operation	Yes
2	Mixing of LARII and lysate	Shaking duration	1.6 s
		Shaking speed	Fast
		Shaking diameter	0.20 mm
		Shaking type	Linear
		Repeated operation	Yes
3	Measurement of firefly luciferase signal	Name of the label	CPS-1s
		Label technology	Luminometry
		Emission filter name	No filter
		Emission filter slot	A7
		Measurement time	1.0 s
		Emission aperture	Normal
4	Measurement of firefly luciferase signal	Name of the label	CPS-1s
		Label technology	Luminometry
		Emission filter name	No filter
		Emission filter slot	A7
		Measurement time	1.0 s
		Emission aperture	Normal
5	Measurement of firefly luciferase signal	Name of the label	CPS-1s
		Label technology	Luminometry
		Emission filter name	No filter
		Emission filter slot	A7
		Measurement time	1.0 s
		Emission aperture	Normal
6	Measurement of firefly luciferase signal	Name of the label	CPS-1s
		Label technology	Luminometry
		Emission filter name	No filter
		Emission filter slot	A7
		Measurement time	1.0 s
		Emission aperture	Normal
7	Measurement of firefly luciferase signal	Name of the label	CPS-1s
		Label technology	Luminometry
		Emission filter name	No filter
		Emission filter slot	A7
		Measurement time	1.0 s
		Emission aperture	Normal
8	Injection of Stop&Glow solution	Injector	2
		Speed	5
		Volume	45 µL
		Increment	0 µL
		Replicate	1
		Injection mode	aspVol=dispVol
		Repeated operation	Yes
9	Mixing of Stop& Glow and lysate	Shaking duration	1.6 s
		Shaking speed	Fast
		Shaking diameter	0.20 mm
		Shaking type	Linear
		Repeated operation	Yes

	Description	Specifications	Values
10	Measurement of Renilla luciferase signal	Name of the label	CPS-1s
		Label technology	Luminometry
		Emission filter name	No filter
		Emission filter slot	A7
		Measurement time	1.0 s
		Emission aperture	Normal

Table 3 VictorLight luminometer program

Data processing

The raw luminescence data was exported from the VictorLight software as Excel-sheets, parsed using a custom-tailored parser written in BASIC and imported into the database. The raw luminescence values were filtered out if they were below an absolute level of 8000 relative units for the *Renilla* luciferase signal and 100 relative units for the firefly signal. The limit for the firefly luciferase signal was 5 times the background signal. One value pair of reporter and control signal was only processed further if both values were above their respective thresholds.

Reporter luciferase signals were corrected against the control luciferase signal within one value pair. To reduce the effect of outliers within the group of triplicates the median was calculated as opposed to the average, see equation 1. The standard deviation was calculated according to equation 2. This value was then normalized against the average of ratios within this 96-well plate of clones to correct for variation between different plates, see equation 3. Standard deviations were normalized with the same value.

The normalized ratios of clones were transformed to log space to allow generating symmetry between repressors and activators. A histogram with a bin size of 0.02 and a range of -1.00 to +2.00 was calculated using the Prism software[8]. A Gaussian normal distribution was fitted to the data (equation 4). The R^2 for the fit was 0.9940, indicating the validity of the mathematical approach as the regression curve fits the data well. However, the runs test shows a significant deviation from the model in the data ($p<0.0001$ in 31 runs) reflecting the fact that a significant number of clones show a more-than-random deviation from the average luminescence ratio. Measured ratios below the first or above the second intersection with the x-axis were considered as

[8] Prism4 for Macintosh, Version 4.0a, GraphPad Software, Inc., San Diego, USA

high-confidence candidates as they are unlikely to be random variations from the mean value, see equation 5(1).

To determine the standard variation of the ratios, the median of all standard deviation was calculated using Prism. Ratios with relative standard deviations larger than the median of all ratios plus the standard deviation of the median were excluded from this high-confidence candidate list; see equation 5(2).

$$ratio(i)_{median} = 10^{\frac{\sum_{n}^{i=1} \log \frac{luc_i}{renilla_i}}{n}}$$

, n = number of assays, luc_i= firefly luciferase signal, $Renilla_i$= Renilla signal

Equation 1 calculation of median ratio firefly over *Renilla*

$$\sigma(i) = \sqrt{\frac{\sum_{n}^{e=1} (ratio(e) - ratio(i)_{median})^2}{n-1}}$$

,σ = standard deviation i = individual clone, n = number of assays, i.e. 2 or 3

Equation 2 Calculation of standard deviation

$$ratio(i)_{corrected} = \frac{ratio(i)_{median}}{\frac{\sum_{n}^{e=1} ratio(e)_{median}}{n}}$$

, i = individual clones, n = number of clones per plate

Equation 3 corrected of median ratio across plate

$y = \frac{A}{\sigma\sqrt{2\pi}} e^{-\frac{(\frac{x-\bar{x}}{\sigma})^2}{2}}$, *A= integral area of graph,* σ *= standard deviation of data, y= value on y-axis for any x,* $\bar{x}$ *=mean of x-values*

Equation 4 Gaussian distribution for ratios

$(1) ratio(i)_{corrected} < threshold_{repressor} \vee ratio(i)_{corrected} > threshold_{activator}$ *,threshold= global thresholds*

$(2) \sigma(i) \leq \tilde{\sigma} + \sigma(\sigma)$ *,* $\sigma(i)$*= standard deviation of clone,* $\tilde{\sigma}$ *=median of all standard deviations,* $\sigma(\sigma)$*= standard deviation of all standard deviations*

Equation 5 Criteria for high-confidence candidates

In situ hybridization robot Intavis InSituPro program specification

	Command	Parameters	Comment
1	SetTempReg	T0 (OFF)	
2	PrimeNeedle	12000	
3	IncubateVT	15 min 150 PTW->Specimen	Wash with PTw
4	IncubateVT	15 min 150 Hyb-mix->Specimen	Wash Hyb.-Mix
5	SetTempReg	T2 (HIGH)	
6	IncubateVT	1 h 150 Hyb-mix->Specimen	Prehybridization
7	IncubateVT	8 h 200 Probe->Specimen	Hybridization
8	IncubateVT	8 min 200 2xSSC/50%FA->Specimen 5x	Posthybwash 1
9	IncubateVT	10 min 200 2xSSC/50%FA->Specimen	Posthybwash 1
10	IncubateVT	8 min 200 2xSSC->Specimen 5x	Posthybwash 2
11	IncubateVT	10 min 200 2xSSC->Specimen	Posthybwash 2
12	IncubateVT	8 min 200 0.2xSSC->Specimen 5x	Posthybwash 3
13	IncubateVT	10 min 150 0.2xSSC->Specimen	Posthybwash 3
14	SetTempReg	T0 (OFF)	
15	Wait	30 min	
16	IncubateVT	10 min 150 PTW->Specimen 2x	PTw
17	IncubateVT	1 h 200 Blocking soln->Specimen	Blocking
18	IncubateVT	4 h 200 Dig antibody->Specimen	DIG antibody
19	IncubateVT	8 min 200 PTW->Specimen 8x	PTw
20	IncubateVT	8 min 200 Prestaining buffer->Specimen 2x	Prestain Buffer
21	Pause		Add staining buffer to position G and press return
22	IncubateVT	8 min 200 Staining buffer->Specimen 2x	Staining buffer
23	PrimeNeedle	12000	
24	SetTempReg	T0 (OFF)	

Table 4 Intavis InSituPro robot program

Vial	Buffer	Volume ml
A	PTW	72.6
A2		
B	Hyb-mix	10.2
C	2xSSC/50%FA	37.6
D	2xSSC	37.6
E	0.2xSSC	36.1
F	Prestaining buffer	13.2
G	Staining buffer	13.2
H		
I		
J		
K		
L	Blocking solution	7.1
M	Dig antibody	7.1
Reservoir		224.0
Basket	Specimen	Probe

Table 5 Loading form for InSitu Robot Volumes indicated are for 30 probes. Positions are named according to standard nomenclature of manufacturer

Transregulation screen database description

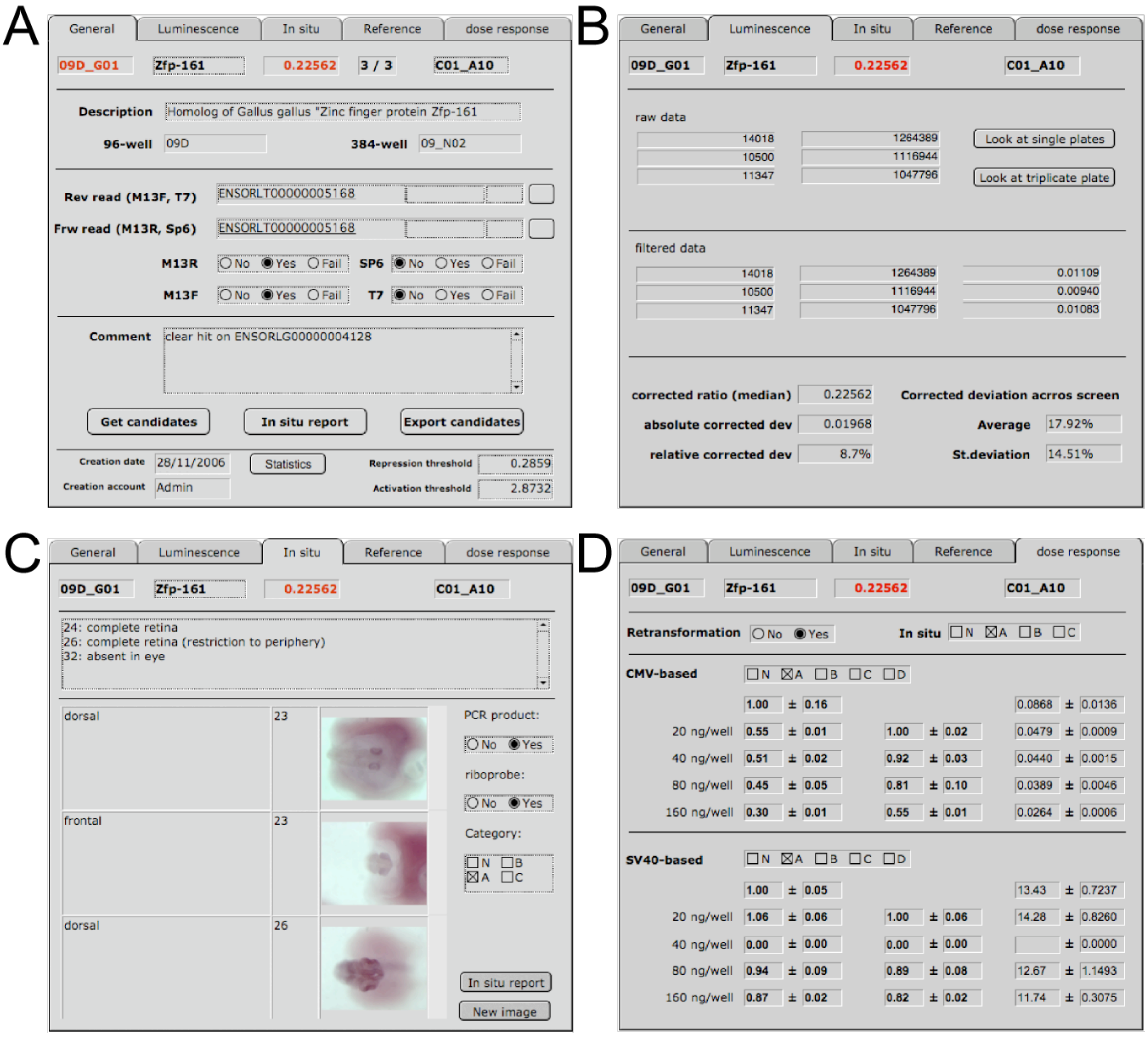

Figure 1 Database Graphical User Interface (GUI) Four of the five existing main GUI displays are depicted here. The user can toggle between the displays using the tab labels at the top of the display. The top section of all displays is the identical. From left to right it contains the clone id, the name of the gene if available, the fold-change in corrected luminescence ration in the screen, the number of successful and total number of assays and a candidate id if applicable. (A) Main display. The first panel from top to bottom contains a short description and the position of the clone in 96- and 384-well format. The second panel gives information about the gene linked to the clone. Information for both forward and reverse read of the clone is available. The radio buttons give information whether the clone has been re-sequenced. The next panel holds a field for comments as well as three buttons for extracting candidates, creating expression pattern reports and exporting candidate clone

information to other programs. The bottommost panel contains administrative information, a link to the statistics page and the global thresholds set for defining candidates. (B) The first panel contains the raw luminescence values and two links to other representations of the raw data. The second panel contains the filtered raw data and the raw ratios. The third panel contains the corrected ratio, the absolute corrected standard deviation and standard deviation in relation to the corrected ratio. It also contains the average of all standard deviations across the screen and their standard deviation. (C) The "In situ" tab contains all information about available expression data. The first panel contains manually annotated expression pattern information. The second panel holds a list of pictures with the orientation and stage of the embryo depicted. The image list can be scrolled and double-click on a picture will open it. The two radio buttons indicate the presence or absence of PCR products and riboprobes for in situ hybridization. Finally, it contains the in situ category. The tab "Reference" is not shown as it only contains a list of referenced files similar to the "In situ tab" (D) This display contains all information about the dose-response curve using CMV::Renilla (second panel) or SV40::Renilla (third panel) as a control. Each of the two panels contains a check box that indicates the summary of the dose-response experiment. In addition the corrected ratios of the different concentrations of regulatory clones together with their standard deviation are shown. The ratio is either normalized against a negative control (first column) or against the lowest concentration of regulator (second column). The third column shows the raw values. The uppermost panel contains some additional practical information about the availability of a retransformed clone.

Generation of fusion proteins

Name	Identifiers		Primer sequence
ELG protein	ENSORLG00000013703	Frw	CCGGAATTCAGC *ATGGAGGTGGAGGAGGGCGAG*
		Rev	CTTGCTCACCATGGC *GGCGTCAAAGTCGTCGCTGCTGT*
NHL protein	ENSORLG00000014595	Frw	CCGGAATTCAGC *ATGCTGCTGCTGAAGAGGAG*
		Rev	CTTGCTCACCATGGC *GACCACGTACGTGCCTAGGCTGAA*
Ndrg3	ENSORLG00000013821	Frw	CCGGAATTCAGC *ATGTCAACTGTTTTGGACCTGGA*

Name	Identifiers		Primer sequence
		Rev	CTTGCTCATCATGAC *ACACGAGACCTCCATGGTCTGA*
SRP40	ENSORLG00000009268	Frw	CCGGAATTCAGC *ATGAGTGGAAGTCGCGTCTTCA*
		Rev	CTTGCTCACCATGGC *GTGTTTGCTGTCTGCCGAGGG*
Zfp161	ENSORLG00000004128	Frw	CCGGAATTCAGC *ATGTCTGACCTACTGAGATACA*
		Rev	CTTGCTCATCATGAC *GCAGTCTCCTGAGTTGTGCGA*
RBPMS2	ENSORLG00000001493	Frw	CCGGAATTCAGC *ATGTATATTAACACGACAGT*
		Rev	CTTGCTCACCATGGC *CTTCAGCTGGACAAAGGTATG*

Table 6 Fusion protein cloning primers
